# NF-κB signaling driven by oncogenic Ras contributes to tumorigenesis in a *Drosophila* carcinoma model

**DOI:** 10.1371/journal.pbio.3002663

**Published:** 2025-04-28

**Authors:** Caroline Dillard, José Teles-Reis, Ashish Jain, Marina Gonçalves Antunes, Paula Ruiz-Duran, Yanyan Qi, Roland Le Borgne, Heinrich Jasper, Tor Erik Rusten

**Affiliations:** 1 Center for Cancer Cell Reprogramming, Institute of Clinical Medicine, Faculty of Medicine, University of Oslo, Oslo, Norway; 2 Department of Molecular Cell Biology, Institute for Cancer Research, Oslo University Hospital, Oslo, Norway; 3 Buck Institute for Research on Aging, Novato, California, United States of America; 4 Univ Rennes, CNRS-UMR, Institut de Génétique et Développement de Rennes, Rennes, France; Fred Hutchinson Cancer Research Center, UNITED STATES OF AMERICA

## Abstract

Cancer-driving mutations synergize with inflammatory stress signaling pathways during carcinogenesis. *Drosophila melanogaster* tumor models are increasingly recognized as models to inform conserved molecular mechanisms of tumorigenesis with both local and systemic effects of cancer. Although initial discoveries of the Toll-NFκB signaling pathway in development and immunity were pioneered in *Drosophila*, limited information is available for its role in cancer progression. Using a well-studied cooperative Ras^V12^-driven epithelial-derived tumor model, we here describe functions of Toll-NF-κB signaling in malignant *Ras*^*V12*^*, scrib*^*-*^ tumors. The extracellular Toll pathway components ModSP and PGRP-SA and intracellular signaling Kinase, Pelle/IRAK, are rate-limiting for tumor growth. The Toll pathway NFκB protein Dorsal as well as *cactus/I*κΒ show elevated expression in tumors with highest expression in invasive cell populations. Oncogenic Ras^V12^, and not loss of *scribble,* confers increased expression and heterogenous distribution of two Dorsal isoforms, DorsalA and DorsalB, in different tumor cell populations. Mechanistic analyses demonstrates that Dorsal, in concert with the BTB-transcription factor Chinmo, drives growth and malignancy by suppressing differentiation, counteracting apoptosis, and promoting invasion of *Ras*^*V12*^*, scrib*^*-*^ tumors.

## Abstract 2

Cancer-driving mutations cooperate with inflammatory cues in cancer progression, but exactly how remains unclear. This study unveils how oncogenic Ras engages inflammation to promote tumor progression in Drosophila melanogaster, showing that activated Toll-NFκB signaling plays an important role in malignant progression and invasion.

## Introduction

Inflammation, defined as the body’s response to harmful external inputs, is a hallmark of cancer [1]. This response is mediated by four major inflammatory pathways: the MAPK, PI3K-AKT, JAK-STAT, and NF-κB (canonical and non-canonical) signaling pathways, which integrate intra- and extracellular alarms and trigger cellular responses [2]. NF-κB activation has been observed in many cancers [3,4] and correlates with a poor prognosis in breast and non-small cell lung cancer [5,6]. NF-κB functions appear cancer-relevant, as a significant body of in vitro studies reports its involvement in cellular processes such as invasion [7–9], proliferation [10,11], stemness [12], survival [10–12], cell competition [13–15], and chemoresistance [12,16–18]. Similarly, a limited number of human cancer xenograft studies demonstrated a positive effect of NF-κB on cancer proliferation [11], survival [11], and metastatic behavior [8,9].

Despite several attempts, pharmaceutical targeting of NF-κB in cancer has proven ineffective due to toxicity resulting from its essential role in regulating cellular and innate immunity at the organismal level [19,20]. Therefore, understanding both the upstream and downstream mechanisms governing NF-κB activation and functions in cancer, as well as deciphering whether such mechanisms are specific to cancer cells, is crucial. This understanding may facilitate the design of NF-κB -targeting cancer therapies that spare healthy cells. In vivo studies would be informative for achieving this goal.

*Drosophila melanogaster* is increasingly being used to study both cancer cell-autonomous mechanisms as well as the biology of complex local and systemic paraneoplastic effects instigated by tumor growth [21]. Among the *D. melanogaster* cancer models, the *Ras*^*V12*^*, scrib*^*-/-*^ carcinoma model, arising from the concomitant expression of the constitute activated oncogenic form of the KRAS/NRAS orthologue, Ras85D (here referred to as Ras^V12^), and the loss of cell polarity through *scribble* loss-of-function, has been most extensively studied and displays a remarkable array of hallmarks similar to human cancer [22,23]. In *Ras*^*V12*^*, scrib*^*-/-*^ tumors, generated in the larval eye-antennal disc (EAD) epithelium, the evolutionarily conserved Ras-MAPK, PI3K-AKT, and JAK-STAT inflammatory pathways all constitute major drivers [22,23]. Surprisingly, at the organismal level, *Ras*^*V12*^*, scrib*^*-/-*^ tumor-bearing flies also display both microenvironmental and systemic alterations that mimic human cancer, such as wasting of adipose and muscle tissues, gut atrophy, microenvironmental extracellular matrix remodeling, and immune cell infiltration [24–27]. Although the discovery and functions of the Toll-NF-κB and IMD-NF-κB pathways were pioneered in flies and found to control developmental patterning and infection response of the innate immune system [28–30], less information is available for its possible roles in carcinogenesis.

Here, we describe the aberrant expression and function of a NF-kB transcription factor in the *Ras*^*V12*^*; scrib*^*-/-*^ tumor model. We demonstrate that in *Ras*^*V12*^*; scrib*^*IR*^ tumors, the NF-kB transcription factor Dorsal (Dl) plays a pro-tumorigenic role by opposing differentiation and promoting survival and invasion. Interestingly, we report the expression of two different splicing variants of Dl, DorsalA (DlA), and DorsalB (DlB), in non-overlapping tumor cell populations. In this context, aberrant Dorsal expression can amplify Chinmo and JNK signaling activation, a known driver of invasion in this model. We also found that *snail* and *twist* genetically contribute to tumor growth.

## Results

### The Toll pathway promotes tumor growth in an autonomous manner

**Fig 1:**
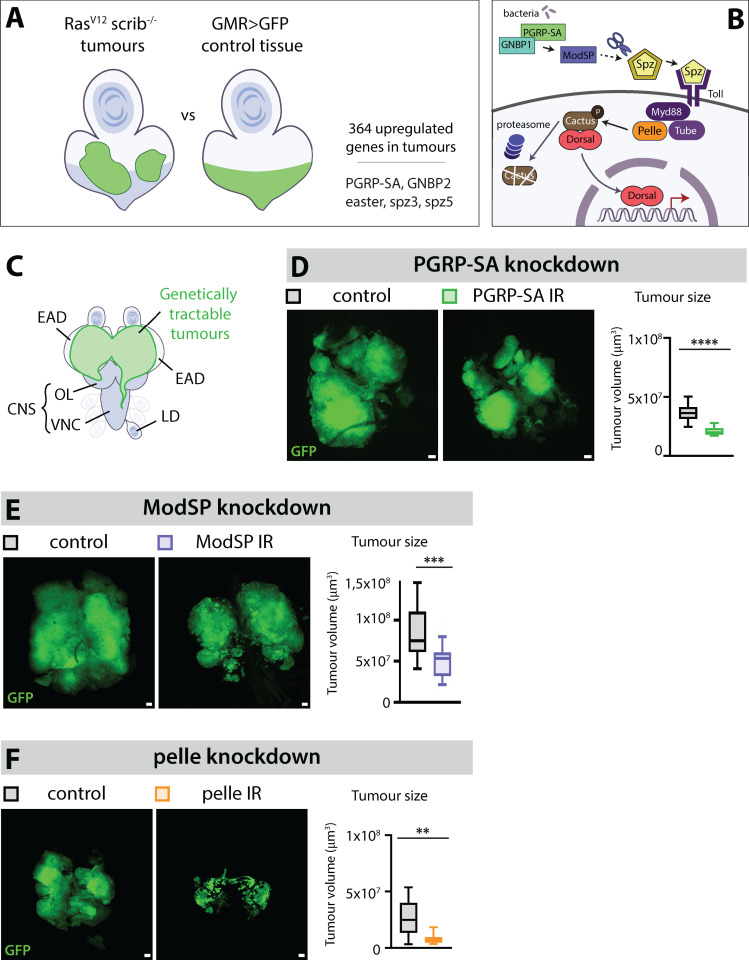
Extracellular and intracellular Toll signaling components contribute to *Ras*^*V12*^*; scrib*^*-/-*^ tumor growth. (A) A transcriptomics analysis comparing the profile of *Ras*^*V12*^*; scrib*^*-/-*^ tumor cells to *GMR>GFP* control cells in the eye-antennal disc (EAD) identified 364 genes that are upregulated in the tumors. Among them, *PGRP-SA*, *GNBP2*, *easter*, *spz3,* and *spz5* are genes taking part in the extracellular machinery leading to Toll pathway activation. PGRP-SA is highlighted in green because it is also found upregulated in two other tumor models. (B) Simplified version of Toll pathway activation upon bacterial infection. PGRP-SA (Peptidoglycan recognition protein SA) is a carboxypeptidase which recognizes and binds to peptidoglycans upon bacterial infection. Follows the formation of a trimeric complex together with a member of the GNBP family (Gram Negative Binding Protein) and the serine protease (ModSP). This complex initiates a multi-step proteolytic cascade that ultimately leads to the cleavage and activation of the ligand Spz. After the binding of Spz to the Toll receptor, the pathway is activated intracellularly through the activation and formation of a complex composed of the adaptor proteins Myd88 and Tube as well as the serine-threonine protein kinase Pelle. Pelle subsequently phosphorylates the inhibitor cactus, the homolog of mammalian IkappaB, which is targeted to proteasome degradation. As Cactus sequesters the NF-kB Dorsal into the cytoplasm, its degradation triggers the translocation of Dorsal into the nucleus and the transcription of its target genes. (C) Cartoon of the genetic setting used in this study for manipulating *Ras*^*V12*^*; scrib*^*-/-*^ tumors. GFP-labeled tumors are generated from randomly selected single epithelial cells from the EAD. They grow and fuse to form large tumors that start invading the central nervous system through the optic lobe first and the ventral nerve cord later. Tumor cells also sometimes reach the leg discs. (D) Representative confocal pictures and quantifications of the mean tumor volumes of GFP-labeled control tumors (*n* = 21, *m* = 3,71 × 10^7^ µm^3^, SD = ±0,47 × 10^7^ µm^3^) and *PGRP-SA Ras*^*V12*^*; scrib*^*-/-*^ tumors (*n* = 17, *m* = 2,11 × 10^7^ µm^3^, SD = ±0,19 × 10^7^ µm^3^) at Day 8 after egg laying, statistical significance was determined with an unpaired *T* test with Welch’s correction. (E) Representative confocal pictures and quantifications of the mean tumor volumes of GFP-labeled control (*n* = 20, *m* = 8,46 × 10^7^ µm^3^, SD = ±1,77,53 × 10^7^ µm^3^) and *ModSP*^*IR*^ *Ras*^*V12*^*; scrib*^*-/-*^ *Dcr2* tumors (*n* = 30, *m* = 5,07 × 10^7^ µm^3^, SD = ±1,00 × 10^7^ µm^3^) at Day 8 (29°C), statistical significance was determined with an unpaired *T* test with Welch’s correction. (F) Representative confocal pictures and quantifications of the mean tumor volumes of GFP-labeled control tumors (*n* = 10, *m* = 2,64 × 10^7^ µm^3^, SD = ±0,76 × 10^7^ µm^3^) and *pelle*^*IR*^ *Ras*^*V12*^*; scrib*^*-/-*^ tumors (*n* = 10, *m* = 0,78 × 10^7^ µm^3^, SD = ±0,22 × 10^7^ µm^3^) at Day 6 (29°C), statistical significance was determined with a Mann–Whitney test. Scale bars = 50 µm.

To identify potential tumor-driving mechanisms, we surveyed upregulated genes in *Ras*^*V12*^*; scrib*^*-/-*^ malignant tumors by RNA Sequencing (Fig 1A). We identified 364 genes with elevated expression levels. These include 14 previously identified genes with defined functions in driving *Drosophila* tumor growth or paraneoplastic effects, such as organ wasting and diuretic dysfunction (S1 and S2 Tables). Notably, among the upregulated genes—PGRP-SA, GNBP2, Easter, Spz3, and Spz5—encode extracellular components of the Toll pathway (Fig 1A). The Toll pathway, one of the two established NF-kB signaling pathways in *Drosophila*, is differentially activated during development and in response to fungal or bacterial infection. PGRP-SA (Peptidoglycan Recognition Protein SA) is a secreted protein that binds to bacteria-derived peptidoglycans upon bacterial infection, forming a trimeric complex with a member of the GNBP family (Gram-Negative Binding Protein) and the serine protease ModSP (Modular Serine Protease). This association initiates a multi-step proteolytic cascade, eventually cleaving the ligand Spz (Spaetzle). Subsequently Spz binds to the Toll receptor, activating the Toll pathway intracellularly (Fig 1B). To date, six Spz ligands and nine Toll receptors have been identified and the functions and associations of the different ligands and receptors are not yet fully understood [30]. Intracellularly, Toll receptor activation triggers the activation and formation of a complex composed of the adaptor proteins Myd88 and Tube as well as the serine-threonine protein kinase Pelle. Pelle subsequently phosphorylates the inhibitor Cactus, the homolog of mammalian IkappaB, which is targeted to proteasome degradation. In the absence of Toll signaling, Cactus sequesters the NF-kB proteins Dorsal or Dif into the cytoplasm. Degradation of Cactus upon Toll signaling triggers the translocation of the NF-kBs into the nucleus with resulting transcription of their target genes (Fig 1B).

We decided to first investigate whether PGRP-SA is involved in *Ras*^*V12*^*; scrib*^*-/-*^ tumorigenesis. To do so, we genetically induced the formation of *Ras*^*V12*^*; scrib*^*-/-*^ tumors from randomly induced single cells in the EAD of the *Drosophila* larva. This way, we can knockdown or overexpress any gene of interest within the tumor specifically without affecting expression in the rest of the body (Fig 1C). To improve RNA interference (IR) expression levels and processing, respectively, knockdown experiments were sometimes performed at 29°C with the additional expression of Dicer-2, as indicated in the legends.

Knock-down of PGRP-SA (*PGRP-SA*^*IR*^) significantly reduced *Ras*^*V12*^*; scrib*^*-/-*^ tumor growth compared with control tumors (Fig 1D). Similarly, knock-down of ModSP (*ModSP*^*IR*^) significantly decreased tumor size (Fig 1E), suggesting that the extracellular components of the Toll pathway surprisingly play a pro-tumorigenic role in this context. To address whether Toll signaling is required within tumor cells or in another microenvironmental compartment, we performed knock-down of the intracellular kinase Pelle/IRAK (*pelle*^*IR*^), a hub in Toll signal transduction. *pelle*^*IR*^ *Ras*^*V12*^*; scrib*^*-/-*^ tumors exhibited a drastic size reduction compared with control tumors (Fig 1F). These results collectively suggest that *Ras*^*V12*^*; scrib*^*-/-*^ tumors produce extracellular Toll-activating components that contribute to a Toll pathway pro-tumorigenic signal activity in an autonomous manner.

### Dorsal is ectopically expressed in oncogenic *Ras*^*V12*^-driven tumors

Toll pathway activation leads to the release and nuclear translocation of the NF-κB transcription factors Dl and/or Dif, leading to transcription of target genes. While most studies of Dl and Dif in developmental patterning and infection have focused on their main splicing variants (DlA and DifA), both NF-kB transcription factors have been reported to also have alternatively spliced B isoforms (DlB and DifB) [31,32]. For instance, both DlA (678 amino acids) and DlB (994 amino acids) contain a Relish-homology domain, composed of a DNA binding domain and a dimerization domain (for homo/heterodimerization and Cactus binding), as well as a transactivation domain (for transcription activation) [31]. However, the transactivation domain of DlA is much shorter than Dorsal-B. Noteworthy, only DlA possesses an NLS sequence. Therefore, DlB cannot translocate to the nucleus by itself but is thought to be able to bind to DlA for translocating to the nucleus of fat body cells upon infection [31]. Contrary to DlA, DlB is not expressed and involved in embryonic dorso-ventral patterning. It is rather specifically expressed in the subsynaptic reticulum of neuromuscular junctions, where it stabilizes Cactus. Similarly, Dif-B is found in the mushroom body of the central nervous system (CNS), where it also stabilizes Cactus [32]. Although these observations support the idea that B isoforms play a specific role in nervous system tissues, their precise functions remain to be elucidated.

**Fig 2:**
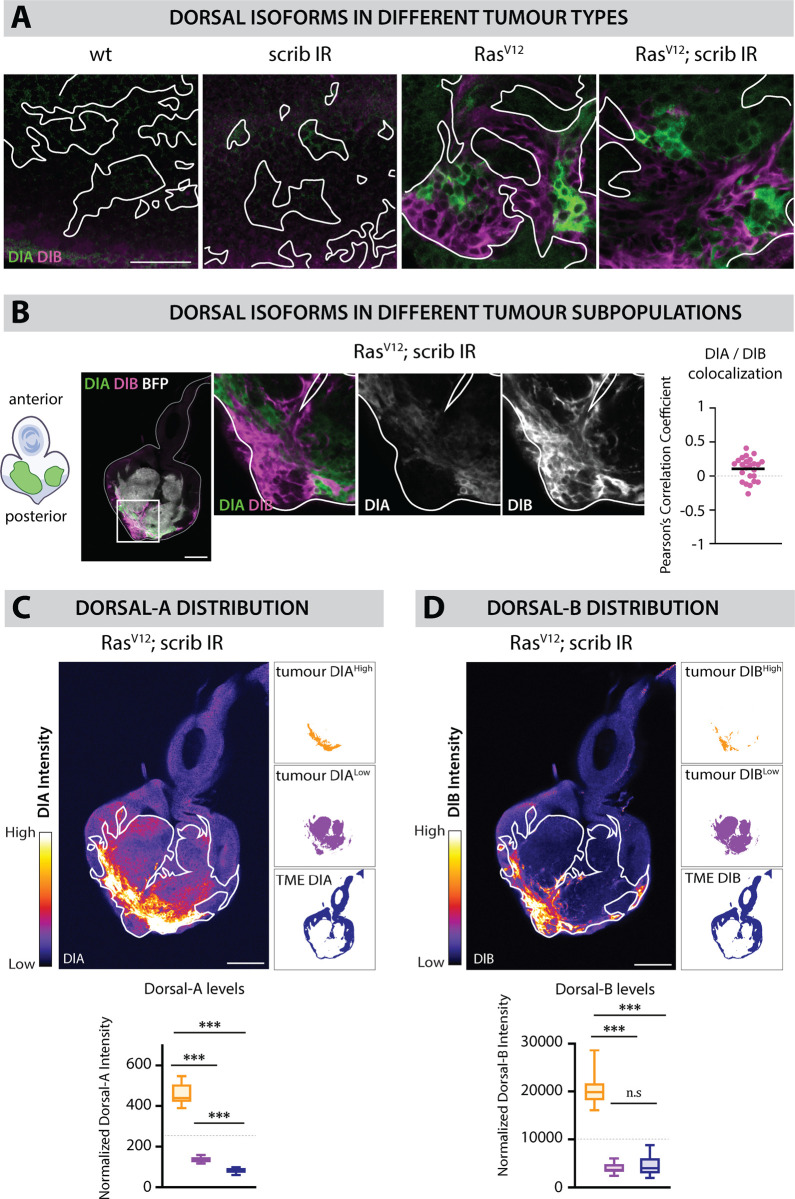
Two splicing variants of the NF-kB Dorsal are aberrantly expressed in *Ras*^*V12*^-driven tumors. (A) Immunostainings against Dorsal-A (DlA in green) and Dorsal-B (DlB in magenta) in BFP-labeled *wt* clones (*n* = 20 EADs), *scrib*^*IR*^ tumors (*n* = 23 EADs), *Ras*^*V12*^ tumors (*n* = 20 EADs), and *Ras*^*V12*^*; scrib*^*IR*^ tumors (*n* = 24 EADs) at Day 6. DlA and DlB accumulation is observed only in *Ras*^*V12*^-driven tumors and appears mainly mutually exclusive. (B) Immunostaining against DlA (green) and DlB (magenta) in BFP-labeled *Ras*^*V12*^*; scrib*^*IR*^ tumors (white) (*n* = 24 EADs) and close-ups showing that DlA and DlB are not expressed in the same subset of cells. Colocalization analysis assessed with the Pearson’s correlation coefficient reveals a poor colocalization between DlA and DlB in the tumors (*n* = 24, *m* = 0,11, SD = ±0,08). (C) Heat map of DlA intensity within a representative BFP-labeled *Ras*^*V12*^*; scrib*^*IR*^ tumor-bearing EAD at Day 6. The tumor is outlined with the straight white line. An arbitrary intensity threshold set at 250 (gray line on the graph) allows the clear segregation of a DlAhigh (“tumor High”) and a DlAlow (“tumor Low”) cell population within the tumor. Quantifications of the mean DlA intensity of the “tumor High” (*n* = 23, *m* = 458,7, SD = ±28,4), “tumor Low” (*n* = 23, *m* = 135,2, SD = ±7,0), and wt tumor microenvironment (TME) (*n* = 23, *m* = 82,2, SD = ±6,7) demonstrate a general elevation of DlA levels within the tumor compared with wt cells. Statistical significance was determined with a Tukey’s multiple comparison test. (D) Heat map of DlB intensity within a representative RasV12; scribIR tumor-bearing EAD at Day 6. The tumor is outlined with the straight white line. An arbitrary intensity threshold set at 10,000 (gray line on the graph) allows the clear segregation of a DlBhigh (“tumor High”) and a DlBlow (“tumor Low”) cell population within the tumor. Quantifications of the mean DlB intensity of the “tumor High” (*n* = 24, *m* = 20,331,9, SD = ±3,108,7), “tumor Low” (*n* = 24, *m* = 3,955,8, SD = ±584,5), and wt TME (*n* = 23, *m* = 4,660,9, SD = ±1,187,3). Normalization was done by subtracting the background intensity. Statistical significance was determined with a Dunn’s multiple comparison test. Scale bars = 20 µm.

We began by assessing the expression of Dl in *Ras*^*V12*^*; scrib*^*IR*^ tumors through immunostainings against DlA and DlB (Figs 2A, 2B, 2C, 2D, S1A, and S1B). Both Dorsal isoforms were highly expressed in tumors in the posterior part of the disc that attaches to the brain through the prospective optic stalk (Fig 2A and 2B), while neither DlA nor DlB proteins were observed in control clones (Fig 2A). DlA and DlB were almost entirely mutually exclusive, in different cell populations (Figs 2A, 2B, and S1B). Within *Ras*^*V12*^*; scrib*^*IR*^ tumors, cells can be easily separated according to their levels of DlA mean intensity (Fig 2C). We identified a DlA^high^ cell population that had 4-fold higher DlA protein levels compared with the DlA^low^ remaining cell population. Importantly, the DlA mean intensity in the DlA^low^ cell population was consistently higher than in the surrounding *wt* epithelial cells of the EAD (Fig 2C). These observations suggest that although we observed a peak of DlA expression at the posterior part of the tumor, there is a general elevation of the basal level of DlA throughout the entire tumor. In contrast, we did not observe a general elevation of DlB throughout the tumor when compared with *wt* cells of the tumor microenvironment (TME), but we did observe a local increase in the posterior part of the tumor of a similar magnitude to DlA (Fig 2B and 2D). Tumor cells located in the posterior area of the tumor are closest to the CNS, which is the first organ these tumors invade [33–35]. Intriguingly, tumor cells that have initiated invasion into the optic lobes (OL) of the CNS displayed high levels of DlA or DlB (S1B Fig), suggestive of a potential role of Toll-NFκB signaling in tumor cell migration.

Finally, a strong enrichment of the repressor Cactus, the ortholog of mammalian IkappaB (IKKB), was observed in DlA^high^ tumor cells but not in DlB^high^ tumor cells, as assessed with an endogenous GFP fusion protein reporter, *Cactus::GFP* (S1C Fig). This is particularly interesting as *cactus* is a positive transcriptional target of the Toll pathway [36]. Cactus upregulation constitutes a negative feedback loop which could cap Toll pathway activation, a mechanism that has also been described in mammalian NF-kB signaling pathways [37,38]. In conclusion, simultaneous elevation of DlA, DlB, and Cactus expression suggests that in the posterior part of *Ras*^*V12*^*; scrib*^*IR*^ tumors, Toll pathway activation is significantly higher than in the rest of the tumor.

### Dorsal aberrant accumulation is genetically and spatiotemporally regulated

**Fig 3:**
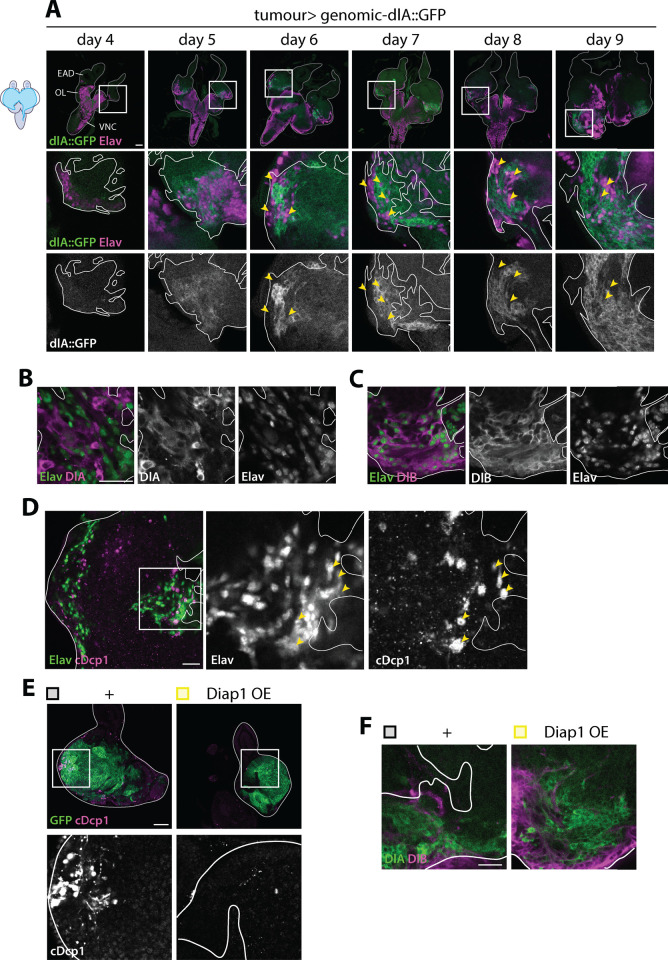
Cell type-dependent Dorsal isoforms accumulation correlates with tumor neurogenesis and apoptosis. (A) Representative confocal pictures of BFP-labeled *Ras*^*V12*^*; scrib*^*IR*^ tumors, expressing a DlA::GFP fusion protein at endogenous levels (green), overtime (Day 4 *n* = 18 EADs, Day 5 *n* = 14 EADs, Day 6 *n* = 18 EADs, Day 7 *n* = 17 EADs, Day 8 *n* = 20 EADs, and Day 9 *n* = 11 EADs) stained for the pan-neuronal marker Elav (magenta). The cephalic complex is outlined with the straight gray line and the tumor is outlined with the straight white line on the close-ups. Yellow arrows point at neurons that are negative for DlA. Scale bar = 50 µm. (B) Representative confocal pictures of GFP-labeled *cherry*^*IR*^ *Ras*^*V12*^*; scrib*^*IR*^ (*n* = 8 EADs) at Day 6 (29°C) stained for Elav (green) and DlA (magenta). The tumor is outlined with the straight white line. DlA and Elav appears mutually exclusive. Scale bar = 20 µm. (C) Representative confocal pictures of GFP-labeled *cherry*^*IR*^ *Ras*^*V12*^*; scrib*^*IR*^ (*n* = 8 EADs) at Day 6 (29°C) stained for Elav (green) and DlB (magenta). The tumor is outlined with the straight white line. DlB and Elav are expressed in the same tumor cells. Scale bar = 20 µm. (D) Representative confocal pictures of GFP-labeled *cherry*^*IR*^ *Ras*^*V12*^*; scrib*^*IR*^ control tumors (*n* = 62 EADs) at Day 6 (29°C) stained for Elav (green) and cDcp1 (magenta). The tumor is outlined with the straight white line. cDcp1^+^ cells are mostly found in areas of the tumor where neurons are present where we often observe neuron apoptosis (yellow arrows). Scale bar = 20 µm. (E) Representative confocal pictures of GFP-labeled *Ras*^*V12*^*; scrib*^*IR*^ control tumors (*n* = 7 EADs) and *Diap1*^*OE*^ *Ras*^*V12*^*; scrib*^*IR*^ (*n* = 9 EADs) stained for cDcp1 (magenta) at Day 6. Scale bar = 50 µm. (F) Representative confocal pictures of GFP-labeled *Ras*^*V12*^*; scrib*^*IR*^ control tumors (*n* = 13 EADs) and *Diap1*^*OE*^ *Ras*^*V12*^*; scrib*^*IR*^ (*n* = 7 EADs) stained for DlA (green) and DlB (magenta) at Day 6. Scale bar = 20 µm.

We investigated what could be the upstream mechanism leading to aberrant Dl accumulation. Initially, we examined Dl isoforms levels in various genetic backgrounds: control *wild type* clones, *scrib*^*IR*^ tumors, *Ras*^*V12*^ tumors, and *Ras*^*V12*^*; scrib*^*IR*^ tumors. We observed aberrant expression of DlA as well as DlB exclusively in *Ras*^*V12*^ and *Ras*^*V12*^*; scrib*^*IR*^ tumors (Fig 2A), suggesting that *Ras*^*V12*^ instructs tumor cells competent to produce these isoforms at abnormal levels. Furthermore, we followed DlA levels over time in *Ras*^*V12*^*; scrib*^*IR*^ tumors, using an endogenous Dorsal genetic reporter, that acts as a Dorsal-A specific reporter in this context (Figs 3A, S2A, and S2B). The onset of DlA expression correlated with the appearance of Elav-positive neurons in the prospective eye region of the tumors around Day5 (Fig 3A). Interestingly, DlA accumulate in cells surrounding the neurons but was rarely detected in the neurons themselves (Fig 3A yellow arrows and Fig 3B). Concurrently, DlB expression was exclusive to the tumor neurons (Fig 3C). Within the tumors, apoptosis was predominantly observed in the differentiating areas where we observed dying neurons (Fig 3D, yellow arrows). Therefore, we hypothesized that the local aberrant Dl expression might be triggered by cell death-derived signals. However, efficient blocking of apoptosis in *Ras*^*V12*^*; scrib*^*IR*^ tumors through Diap1 overexpression (*Diap1*^*OE*^) did not affect increased DlA and DlB levels (Fig 3E and 3F).

Altogether, these observations demonstrate that the aberrant expression NF-kB/Dl in *Ras*^*V12*^*; scrib*^*IR*^ tumors is both genetically and locally controlled. General elevated levels of DlA in the whole tumor populaton suggest a transcriptional response downstream of oncogenic Ras^V12^ signaling. On top of this Ras^V12^-dependent genetic control, a temporal and/or local control triggers elevated levels of DlA, DlB, and Cactus specifically in the posterior part of the tumor in the prospective eye region, as well as in invading tumor cells to the brain.

### Dorsal represses Ras^V12^ scrib^IR^ tumor differentiation and death

**Fig 4:**
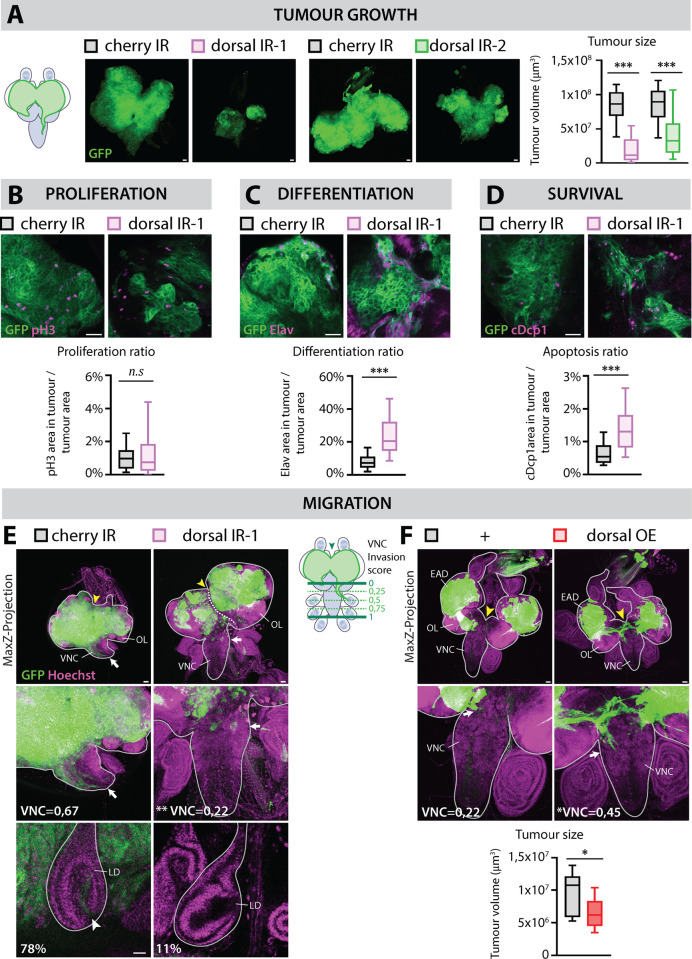
Dorsal represses differentiation and apoptosis in *Ras*^*V12*^ *scrib*^*RNAi*^ tumors while promoting invasive behavior. (A) Representative confocal pictures and quantifications of the mean tumor volumes of GFP-labeled *Cherry*^*IR*^ *Ras*^*V12*^*; scrib*^*IR*^ control tumors (*n* = 36, *m* = 8,32 × 10^7^ µm^3^, SD = ±1,31 × 10^7^ µm^3^), *dorsal*^*IR-1*^ *Ras*^*V12*^*; scrib*^*IR*^ tumors (*n* = 25, *m* = 1,97 × 10^7^ µm^3^, SD = ±1,00 × 10^7^ µm^3^), *Cherry*^*IR*^ *Ras*^*V12*^*; scrib*^*IR*^ *Dcr2* control tumors (*n* = 43, *m* = 8,52 × 10^7^ µm^3^, SD = ±1,64 × 10^7^ µm^3^), and *dorsal*^*IR-2*^ *Ras*^*V12*^*; scrib*^*IR*^ *Dcr2* tumors (*n* = 37, *m* = 4,71 × 10^7^ µm^3^, SD = ±1,85 × 10^7^ µm^3^) at Day 12 (29°C), statistical significance was determined with an unpaired *T* test with Welch’s correction. Scale bar = 50 µm. (B) Representative confocal pictures and quantifications of the proliferation ratio of GFP-labeled *Cherry*^*IR*^ *Ras*^*V12*^*; scrib*^*IR*^ control tumors (*n* = 36, *m* = 1,08%, SD = ±0,41%) and *dorsal*^*IR-1*^ *Ras*^*V12*^*; scrib*^*IR*^ tumors (*n* = 35, *m* = 1,39%, SD = ±0,83%) at Day 8 (29°C). Proliferation was detected through immunostainings against the mitotic marker pH3 (phospho-Histone 3, in magenta). Here, the proliferation ratio is defined as the ratio between the pH3 area within the tumor and the area of the tumor itself. Scale bar = 20 µm. (C) Representative confocal pictures and quantifications of the differentiation ratio of GFP-labeled *Cherry*^*IR*^ *Ras*^*V12*^*; scrib*^*IR*^ control tumors (*n* = 63, *m* = 8,29%, SD = ±2,87%) and *dorsal*^*IR-1*^ *Ras*^*V12*^*; scrib*^*IR*^ tumors (*n* = 57, *m* = 24,40%, SD = ±7,09%) at Day 8 (29°C). Differentiation was detected through immunostainings against the pan-neuronal marker Elav (magenta). Here, the differentiation ratio is defined as the ratio between the Elav area within the tumor and the area of the tumor itself. Scale bar = 20 µm. (D) Representative confocal pictures and quantifications of the apoptosis ratio of GFP-labeled *Cherry*^*IR*^ *Ras*^*V12*^*; scrib*^*IR*^ control tumors (*n* = 63, *m* = 0,71%, SD = ±0,29%) and *dorsal*^*IR-1*^ *Ras*^*V12*^*; scrib*^*IR*^ tumors (*n* = 57, *m* = 1,45%, SD = ±0,46%) at Day 8 (29°C). Apoptosis was detected through immunostainings against the cleaved effector caspase, cDcp1 (cleaved-Death Caspase 1, in magenta). Here, the apoptosis ratio is defined as the ratio between the cDcp1 area within the tumor and the area of the tumor itself. Statistical significance was determined with an unpaired *T* test with Welch’s correction. Scale bar = 20 µm. (E) Z-projections of representative confocal pictures of size-matched GFP-labeled *Cherry*^*IR*^ *Ras*^*V12*^*; scrib*^*IR*^ tumors (*n* = 3 EADs) and *dorsal*^*IR-1*^ *Ras*^*V12*^*; scrib*^*IR*^ tumors (*n* = 8 EADs) at Day 12 (29°C), stained for Hoechst (magenta). Scale bars = 50 µm. The cephalic complexes (and leg discs [LD] on the small inserts) are outlined with the straight gray line. A cartoon represents the three invasion parameters assessed in this study: the VNC (Ventral Nerve Cord) invasion score, ranged between c and 1, is greater the further tumor cells migrate toward the tip of the VNC (green arrow), indicated as “VNC=” on the Z-projection images; the frequency of LD invasion (small insert with LD confocal pictures, Scale bars = 20 µm) and the fusion of distinct Eye-Antennal Disc (EAD) tumors (white arrow, dashed line indicates the visible separation of both neighbor EADs). For the VNC score, statistical significance was determined with Dunnett’s T3 multiple comparisons test. (F) Z-projections of representative confocal pictures of GFP-labeled *Ras*^*V12*^*; scrib*^*IR*^ (*n* = 15 EADs) and *dorsal*^*OE*^ *Ras*^*V12*^*; scrib*^*IR*^ tumors (*n* = 24 EADs) at Day 6, stained for Hoechst (magenta). The cephalic complexes (and LD on the small inserts) are outlined with the straight gray line. Quantification of the mean tumor volumes of GFP-labeled *Ras*^*V12*^*; scrib*^*IR*^ (*n* = 9, *m* = 9,71 × 10^6^ µm^3^, SD = ±1,53 × 10^6^ µm^3^) and *dorsal*^*OE*^ *Ras*^*V12*^*; scrib*^*IR*^ tumors (*n* = 11, *m* = 6,52 × 10^6^ µm^3^, SD = ±1,12 × 10^6^ µm^3^) at Day 6. OL: Optic Lobe. Statistical significance was determined with an unpaired *T* test with Welch’s correction. Scale bars = 50 µm.

To investigate the primary functions of Dl in *Ras*^*V12*^*; scrib*^*IR*^ tumors, we conducted knockdown experiments using two independent RNAis that efficiently target both Dorsal isoforms (S3A and S3B Fig). *Dorsal*^*IR*^*, Ras*^*V12*^*; scrib*^*IR*^ tumors exhibited a significant reduction in size compared with *Cherry*^*IR*^ *Ras*^*V12*^*; scrib*^*IR*^ control tumors (Fig 4A), mimicking the phenotypes observed upon *PGRP-SA*, *ModSP,* and *pelle* knockdowns (Fig 1C, 1D, and 1E). *Ras*^*V12*^*; scrib*^*IR*^ tumor growth results from a combination of cell growth, cell proliferation, inhibition of the differentiation of epithelial tumor cells into Elav-positive photoneurons, and survival [33,39]. To delineate the potential functions of Dl in *Ras*^*V12*^*; scrib*^*IR*^ tumors, we assessed alterations in proliferation, differentiation, and apoptosis. Quantification of mitotic cells suggests that *Dorsal*^*IR*^ *Ras*^*V12*^*; scrib*^*IR*^ tumors display similar proliferation rates to control tumors, but both their differentiation and apoptosis rates were significantly higher than in control tumors (Fig 4B, 4C, and 4D). Although, a few neurons form in *Ras*^*V12*^*; scrib*^*IR*^ tumors, they appeared spaced apart and did not form the full complement of eight neurons per ommatidia. However, upon Dorsal knockdown, Elav-positive cells increased in number and density, suggesting a partial rescue of differentiation (Fig 4C). To address how Dorsal may affect differentiation and apoptosis, we induced Dorsal in random clones in the eye antennal disc that can arise in undifferentiated cells ahead of the morphogenetic furrow, or post-furrow, where differentiation of neurons and support cells occur. Select post-furrow expression was achieved using the GMR-GAL4 driver. Whereas large Dorsal-expressing clones straddling the pre- and post-furrow region strongly reduced neuronal differentiation, the expression post furrow did not (S4C Fig). This suggests that Dorsal may inhibit differentiation autonomously only when induced early, either through a strictly cell-autonomous effect or indirectly interfering with the complex cell signaling pathways that drive furrow progression and differentiation. During eye differentiation, supernumerary cells surrounding neurons of each ommatidium are culled through apoptosis in the late larval and pupal stages. Selective post-furrow expression of Dorsal significantly repressed apoptosis in late larval eye discs (S4D Fig) Additionally, the adult eye presented features of enlarged ommatidia consistent with phenotypes of super-numerary cells.

Importantly, *Pelle*^*IR*^ *Ras*^*V12*^*; scrib*^*-/-*^ tumors also exhibited increased apoptosis and differentiation rates (S3C Fig). This emphasizes the need for the Toll signaling pathway and not an alternative way to activate Dorsal/NFkB activity, to promote tumor growth. Thus, the Toll-Dl signaling pathway promotes tumor growth primarily by preventing differentiation and promoting survival.

### Dorsal is required for invasion and accentuates JNK signaling

**Fig 5:**
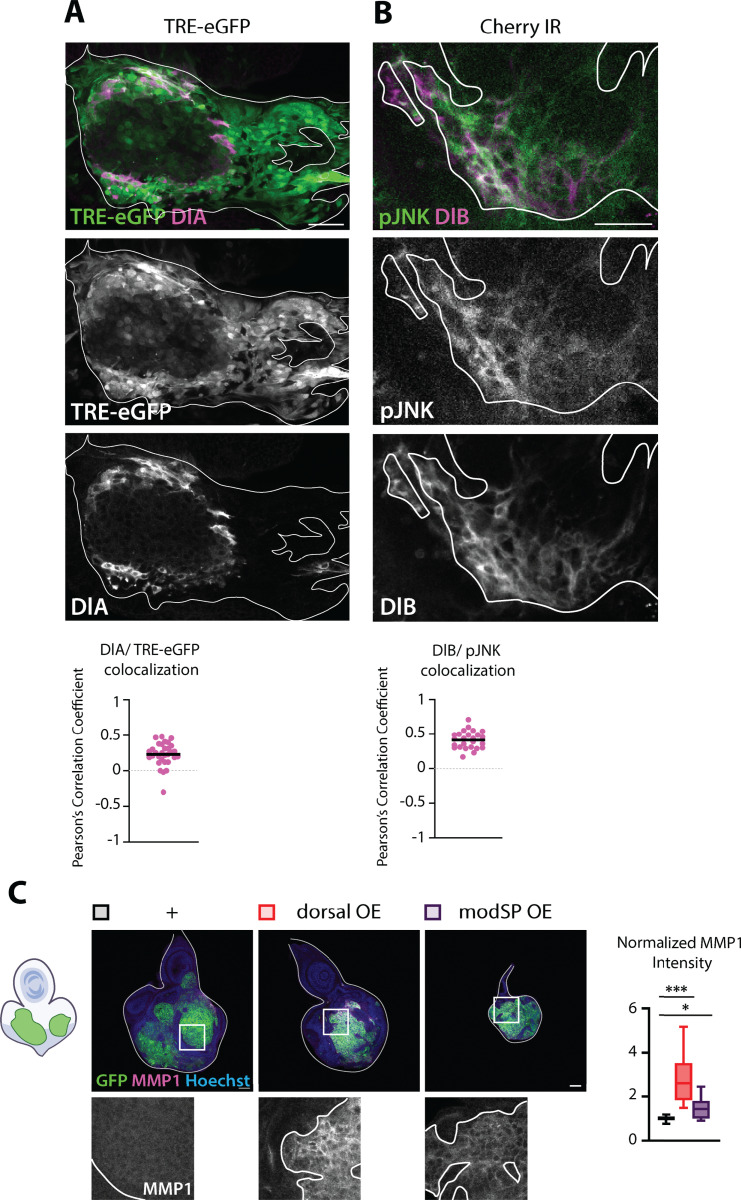
Dorsal amplifies JNK signaling. (A) Representative confocal pictures of BFP-labeled *Ras*^*V12*^*; scrib*^*IR*^ tumors, expressing the JNK activity reporter, TRE-eGFP (green) and stained for DlA (magenta). The tumor is outlined with the straight white line. Colocalization analysis assessed with the Pearson’s correlation coefficient reveals a weak colocalization between DlA and JNK activity in the tumors (*n* = 31, *m* = 0,23, SD = ±0,08). Scale bar = 20 µm. (B) Representative confocal pictures of GFP-labeled *cherry*^*IR*^ *Ras*^*V12*^*; scrib*^*IR*^ tumors stained for pJNK (green) and DlB (magenta) at Day 6 (29°C). The tumor is outlined with the straight white line. Colocalization analysis assessed with the Pearson’s correlation coefficient reveals a good colocalization between DlB and pJNK in the tumors (*n* = 25, *m* = 0,41, SD = ±0,06). Scale bar = 20 µm. (C) Representative confocal pictures and quantifications of the mean MMP1 intensity of GFP-labeled *Ras*^*V12*^*; scrib*^*IR*^ tumors (*n* = 11, *m* = 1,00, SD = ±0,06), *dorsal*^*OE*^ *Ras*^*V12*^*; scrib*^*IR*^ tumors (*n* = 11, *m* = 2,85, SD = 0,57), and *modSP*^*OE*^ *Ras*^*V12*^*; scrib*^*IR*^ tumors (*n* = 10, *m* = 1,49, SD = ±0,32) at Day 6. Intensity normalization was done by subtracting the background intensity on each picture and finally dividing each data point by the mean intensity value for the control tumors for each replicate. Statistical significance was determined with Dunnett’s T3 multiple comparisons test. Scale bars = 50 µm.

We next addressed whether Dl is involved in tumor invasion. In the *Ras*^*V12*^*; scrib*^*IR*^ tumor model, cells adopt a migratory behavior by JNK signaling. Specifically, JNK mediates expression of Matrix MetalloProteinase 1 (MMP1) and Cheerio (Cher-mammalian Filamin), resulting in basement membrane degradation and cytoskeleton remodeling, respectively [25,34,39–43]. We conducted colocalization analyses, assessed by the Pearson’s Correlation Coefficient (PCC), between the Dorsal isoforms and JNK activity in the tumors, assessed either through activated JNK (phosphor-JNK, pJNK) or a JNK activity reporter, TRE-eGFP. Overall, we found a low colocalization between DlA and TRE-eGFP (Fig 5A), while we observed a good PCC between DlB and pJNK within the tumors (Fig 5B). These observations align with the finding that tumor cells invading the CNS often have high levels of either DlA or DlB.

To test whether Dorsal may function upstream of JNK signaling in *Ras*^*V12*^*; scrib*^*IR*^ tumors, we assessed the levels of the JNK target MMP1 following *Dorsal*^*OE*^ and *ModSP*^*OE*^ overexpression which enhances Toll signaling and subsequently Dorsal activity. MMP1 mean intensity was significantly higher in tumors with elevated *Dorsal* or *ModSP* expression compared with control tumors. Therefore, Toll-Dl signaling can augment expression of the JNK transcriptional target, *mmp1* in *Ras*^*V12*^*; scrib*^*IR*^ tumors (Fig 5C).

As both DlA, DlB and Cactus are highly expressed in brain lobe proximal and invading cells and Dl amplifies JNK signaling in the tumors, we investigated the effect of Dorsal knock-down on tumor cell migration. We scored for three parameters reflecting the mobility of tumor cells at late stages of tumor development (Day 12): (1) the VNC invasion score, which assigns a higher score to tumors with greater migration toward the tip of the ventral nerve cord (VNC) of the CNS, (2) the frequency at which we observe tumor cells within the organs adjacent to the VNC called leg discs (LD), and (3) the fusion of tumors generated in the two distinct EAD, indicating the ease with which cells move from one EAD to the other and EADs and come into contact with each other (Fig 4E). To mitigate the influence of tumor cell number on migration assessment, we compared control and *Dorsal*^*IR*^ tumors of similar size. Upon Dorsal knock-down, at late stages, the three parameters showed a drastic decrease: the VNC score decreased 3-fold compared with control, reaching a low value similar to early-stage control tumors (Fig 4F). The frequency of invasion into LD decreased 7-fold and tumors from neighboring EADs often failed to merge (Fig 4A). Similarly, Dorsal overexpression (*Dorsal*^*OE*^) increased tumor cell mobility even at early stages (Day 6), while tumor size is signicantly smaller (Fig 4F), suggesting that (1) the increase in invasion is not simply a secondary consequence of higher cell number and (2) that the balance between invasion and tumor growth tilted toward invasion when tumor cells have high levels of Dorsal.

Given the functions of Dorsal in the tumor context, we wondered whether *Dorsal*^*OE*^ is sufficient to induce tumorigenesis. Although *Dorsal*^*OE*^ control clones were not bigger than *wt* clones, we systematically observed the formation of dysplastic GFP-labeled *Dorsal*^*OE*^ clones within the EADs that appeared as large-rounded cell masses often located basally (S4A and S4B Fig, yellow arrow). We hypothesize that they arise from the inhibition of differentiation. Moreover, in this context, we never observed *Dorsal*^*OE*^ cells in the VNC (VNC score in S4A Fig). Thus, Dorsal at high levels is sufficient to trigger dysplasia but not invasive hyperplastic tumors.

The epithelial-mesenchymal transition (EMT) transcription factor Snail has been implicated in promoting invasion of *Ras*^*V12*^*; lgl*^*-/-*^ tumors (a similar tumor model to *Ras*^*V12*^*; scrib*^*-/-*^ tumors) through stimulation of JNK signaling and EMT-like cytoskeleton remodeling [44]. Snail, along with Twist, another EMT transcription factor, are well-known targets of Dorsal that govern mesoderm invagination during developmental patterning in cells with the highest Dorsal activity [45–48]. Interestingly, knockdowns of both *snail* and *twist* significantly decreased *Ras*^*V12*^*; scrib*^*IR*^ tumor size (S5A and S5B Fig), mimicking *Dorsal* knockdown. At late stages, *twistIR Ras*^*V12*^*; scrib*^*IR*^ tumor also displayed a lower VNC score compared with control tumors, suggesting impaired tumor cell migration (S5B Fig).

### Dorsal and Chinmo can mutually engage each other, prevent differentiation, and promote tumor growth

**Fig 6:**
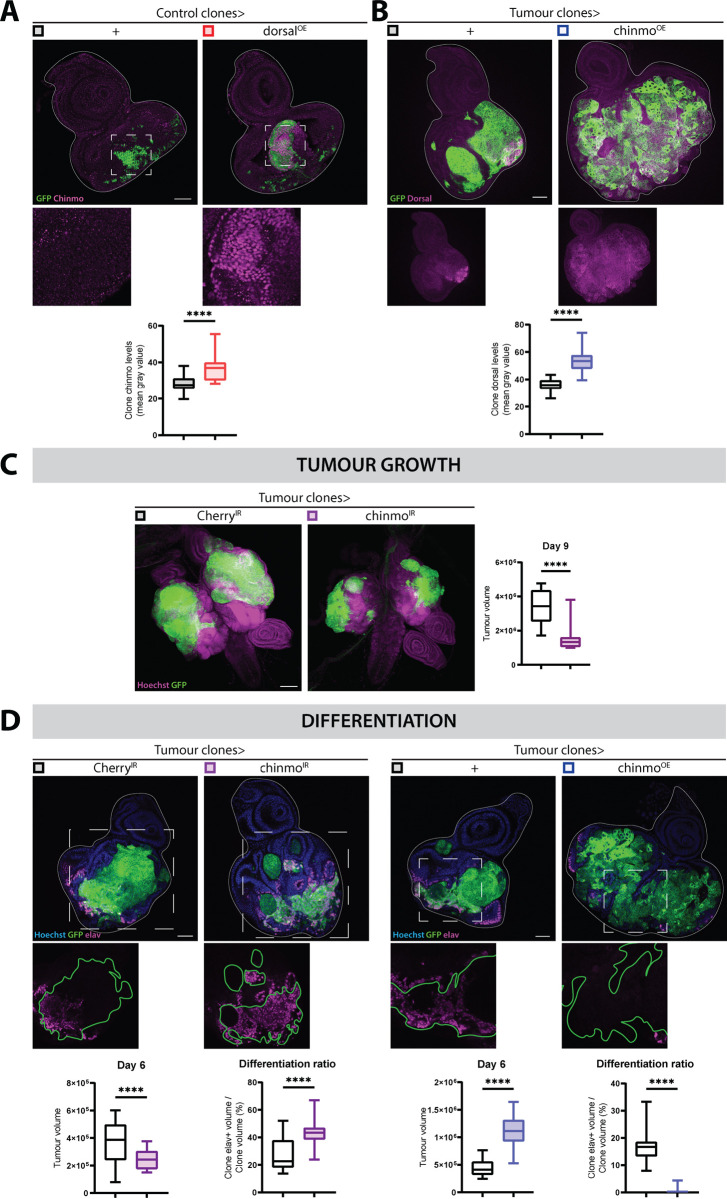
Dorsal and chinmo mutually influence each other’s expression, driving tumor growth while inhibiting differentiation. (A) Representative confocal pictures and quantifications of control clone chinmo levels (mean gray value) of GFP-labeled *wt* clones (*n* = 18, *m* = 28,39, SD = ±4,129) and *dorsal*^*OE*^ clones (*n* = 19, *m* = 36,66, SD = ±6,701) at Day 6. Chinmo (magenta) upregulation upon Dorsal overexpression in clones is visible in the insets bellow each image. Statistical significance was determined with an Unpaired *t* test with Welch’s correction. Scale bar = 50 µm. (B) Representative confocal images and quantifications of clone dorsal levels (mean gray value) of GFP-labeled *Ras*^*V12*^*; scrib*^*IR*^ tumors (*n* = 34, *m* = 36,09, SD = ± 4,009) and chinmo^OE^ *Ras*^*V12*^*; scrib*^*IR*^ tumors at Day 6 (*n* = 45, *m* = 53,93, SD = ± 7,683). Single channel image below shows a clear upregulation of Dorsal (magenta) upon chinmo overexpression in *Ras*^*V12*^*; scrib*^*IR*^ tumors. Statistical significance was identified with an Unpaired *t* test with Welch’s correction. Scale bar = 50 µm. (C) Representative confocal pictures and quantifications of tumor volumetrics for GFP-labeled *Cherry*^*IR*^ *Ras*^*V12*^*; scrib*^*IR*^ tumors (*n* = 17, *m* = 3,429,819 µm^3^, SD = ±942,847 µm^3^) and *chinmo*^*IR*^ *Ras*^*V12*^*; scrib*^*IR*^ tumors (*n* = 11, *m* = 1,502,713 µm^3^, SD = ±797,983 µm^3^) at Day 9. The cephalic complex is counterstained with Hoechst (magenta). Statistical significance was assessed with a Mann–Whitney test. Scale bar = 100 µm. (D) Representative confocal images and quantifications of tumor volumetrics and clone elav+ volume coverage for GFP-labeled (left) *Cherry*^*IR*^ *Ras*^*V12*^*; scrib*^*IR*^ tumors (Volumetrics: (*n* = 30, *m* = 376,663 µm^3^, SD = ±142,575 µm^3^); Elav+ coverage: (*n* = 30, *m* = 27,28, SD = ±12,19)) and *chinmo*^*IR*^ *Ras*^*V12*^*; scrib*^*IR*^ tumors (Volumetrics: (*n* = 25, *m* = 247,329 µm^3^), SD = ±69,250 µm^3^; Elav+ coverage: (*n* = 25, *m* = 44,25, SD = ±10,19)). Statistical significance for volumetrics was identified with an Unpaired *t* test with Welch’s correction and a Mann–Whitney test was used for clone elav+ coverage quantifications. Insets reveal an increase of elav+ (magenta) cells within the tumor upon chinmo^IR^; (right) *Ras*^*V12*^*; scrib*^*IR*^ tumors (Volumetrics: (*n* = 34, *m* = 439,561 µm^3^, SD = ±133,270 µm^3^); Elav+ coverage: (*n* = 44, *m* = 16,70, SD = ± 4,944)) and *chinmo*^*OE*^ *Ras*^*V12*^*; scrib*^*IR*^ tumors (Volumetrics: (*n* = 45, *m* = 1,118,657 µm^3^, SD = ± 247,752 µm^3^); Elav+ coverage: (*n* = 51, *m* = 0,4,118, SD = ± 0,6,376)). Statistical significance for volumetrics was identified with an Unpaired *t* test with Welch’s correction and a Mann–Whitney test was used for clone elav+ coverage quantifications. Insets show a decrease of elav+ (magenta) cells within the tumor upon chinmo^OE^. EAD nuclei were counterstained with Hoechst (blue). Scale bars = 50 µm.

The BTB transcription factor, Chinmo, has previously been found to be induced and repress differentiation in Ras and Raf-driven fly tumor models [43,49]. We therefore investigated the possible interrelationship between Dorsal and Chinmo. Large Dorsal-expressing clones consistently induced high Chinmo expression (Fig 6A). Conversely, Chinmo overexpression can further elevate Dorsal levels in *Ras*^*V12*^*; scrib*^*IR*^ tumors (Fig 6B). We noticed that similar to Dorsal-expressing clones (S4A,S4B), Chinmo expression alone, led to basal epithelial cell delamination and a failure of neuronal differentiation (S6A,S6B). Previous findings have demonstrated that Chinmo expression alone or in the context of Raf^GOF^ expression can effectively repress differentiation of the eye region of the EAD [44]. Consistent with these findings, we observe that further elevating Chinmo in *Ras*^*V12*^*; scrib*^*IR*^ tumors led to a complete block in neuronal differentiation (Fig 6B). We next addressed the potential role of Chinmo in *Ras*^*V12*^*; scrib*^*IR*^ tumors. Chinmo knockdown, effectively reduced Chinmo expression and strongly reduced tumor growth (S6C, and 6C Figs). Strikingly, knockdown of Chinmo almost completely reversed the lack of neuronal differentiation in *Ras*^*V12*^*; scrib*^*IR*^ *tumor*s. These experiments suggest that Dorsal promotes tumor growth through repressing cell death and differentiation as well as promoting EMT-like delamination and invasion in conjunction with Chinmo.

**Fig 7:**
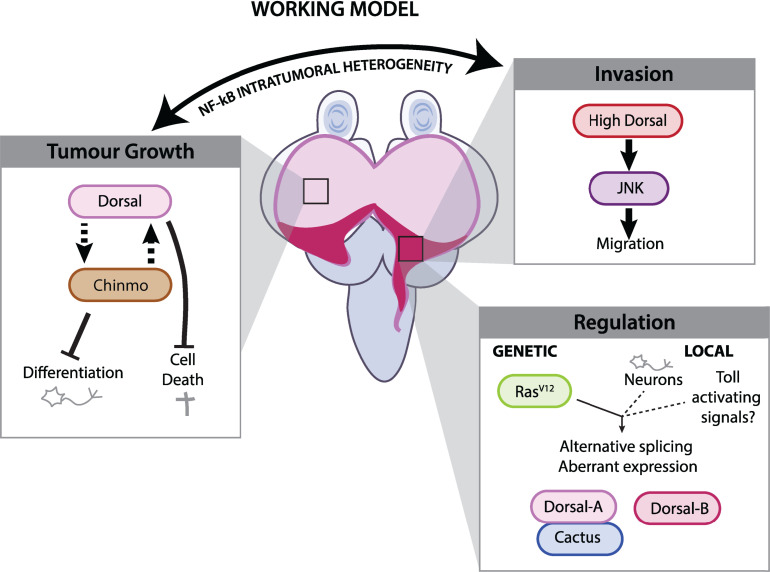
Working model.

We propose a model where the Dl^high^ tumor cells can stimulate JNK and Chinmo that in concert promotes tumor growth and invasion, in part through inhibition of differentiation and apoptosis (Fig 7A).

In this working model, we propose that the protein level of the NF-kB Dorsal regulates different aspect of tumorigenesis. Invasion: In the most posterior part of the tumor, high levels of Dorsal promote invasion of the tumor cells in the adjacent organs through elevation of JNK signaling. Tumor growth: In the rest of the tumor where more intermediate levels of Dorsal are observed, Dorsal would stimulate tumor growth by repressing differentiation and apoptosis in concert with Chinmo. Regulation: At least, two factors seem to trigger aberrant levels of Dorsal within the tumors. Genetically, Ras^V12^ make tumor cells competent to accumulate higher levels of Dorsal throughout the entire tumor. Locally, a spatiotemporal control is exerted at the posterior part of the tumor where very high levels of Dorsal are reached. A possibility is an increase local Toll pathway activation triggered by high Spz concentration from the brain which will promote Dorsal expression. There, we observe alternative splicing of DlA versus DlB which segregate in different tumor subpopulations. We hypothesize that the heterogeneity in Dorsal isoforms and/or levels promote a balance between tumor growth and invasion favoring tumorigenesis.

## Discussion

Here, we establish that autonomous activation of the inflammatory Toll pathway plays a pivotal role in supporting the growth of *Ras*^*V12*^*; scrib*^*IR*^ tumors. The NF-kB transcription factor Dorsal, and key effectors of the Toll pathway, exhibit aberrant expression in this model. In this context, Dorsal activity facilitates tumor growth by preventing differentiation and promoting survival without measurable impact on proliferation. Additionally, Dorsal promotes invasion genetically dependent on the EMT factors Snail and Twist.

An exciting feature in this tumor model is tumor heterogeneity in both NF-kB protein composition (DlA and DlB) and Toll pathway activity (Dl^high^ cactus^high^ versus Dl^low^, Cactus^low^), as judged by classical target gene expression (*cactus* and *dorsal*) and stimulation of invasion. Dl is unevenly accumulated within the population of transformed cells in a spatial and regional manner and on at least two levels: there is a low-level DlA accumulation within the entire region of Ras^V12^ expression and a heightened level in the posterior region situated in the differentiating prospective eye region that is physically attached to the brain through the stalk. In this region, a notable accumulation of both splicing variants of Dl, DlA, and DlB is seen within distinct tumor cell populations.

While NF-kB protein heterogeneity within the same tumor has not been studied yet in solid mammal tumors, the NF-kB fingerprinting and their subsequent homo-/heterodimerization within diffuse large B cell lymphoma hold predictive values for the tumor cell response to microenvironmental activating cues [50]. With our study, this finding suggests that NF-kB composition heterogeneity might also occur in solid mammalian cancers and modulate their response to microenvironmental inflammation.

Moreover, heterogeneity in the level/activity of a single NF-kB mirrors observations in mammalian cancers. In pancreatic cancer, where oncogenic KRAS is the main driver, tumor heterogeneity is a central hallmark and frequently associated with variable NF-kB signaling. A central question is thus, what causes some cells to respond with inflammation whereas others do not? Possibilities include non-autonomous external input from nearby cells of the microenvironment or circulation versus intrinsic mechanisms. In vitro studies of KRAS^G12V^-transfected immortalized human pancreatic epithelial derived cells have demonstrated that variable levels of oncogenic KRAS can produce different outcomes. High KRAS^G12V^ expression can direct NF-kB signaling and EMT phenotypes driven in part by feed-forward loop through IL6 signaling, whereas lower level of KRAS activity retains epithelial character with lower level of NF-kB signaling dependent on IL1 [51].

In the *Ras*^*V12*^*, scrib*^*-/-*^ model, a “flip out” cassette turns on Ras^V12^ expression under control of the actin promoter in all cells with the same genetic dose, and by first principle should signal evenly. Yet, Ras^V12^-driven NF-kB expression is temporally and spatially controlled in a stereotypic pattern. One possibility is that this region is prepatterned to respond to increased levels of Ras^V12^ with a higher response. Indeed, in this region, we observe a higher level of pMAPK and where tumor region neurogenesis is incomplete as we do not observe fully formed ommatidia emphasizing the inhibitory effect of Dorsal on differentiation. In the prospective eye region, behind the morphogenetic furrow, founder R8 neurons recruit further neurons through Receptor Tyrosine Kinase-MAPK signaling that potentially could add up with Ras^V12^-MAPK signaling to reach a higher signaling levels [52].

Extrinsic regulation of Toll signaling does have a role in the *Ras*^*V12*^*, scrib*^*-/-*^ *t*umor model for growth. The Toll-activating components implicated in tumor growth, PGRP-SA and ModSP, are part of a bacterial recognition alert system. The observed overexpression of PGRP-SA in *Ras*^*V12*^; *scrib*^*IR*^ tumors may serve to locally increase the concentration of active cleaved Spz ligands, thereby stimulating Toll activation and promoting tumor growth. The source of activation governed by PGRP-SA and ModSP remains unknown. As the posterior part of the disc experience higher levels of apoptosis, we considered whether apoptotic cell death and debris could active PGRP-SA/ModSP—driven Toll sigaling in this context through death—induced danger signals and bacterial component mimicry, but inhibition of death through DIAP1 expression did not alter NFkB levels.

Notably, previous studies have identified PGRP-SA, Cactus, and Dorsal as positive targets of the Toll pathway [36]. Furthermore, it has been shown that the CNS serves as a significant source of Spz ligands that regulates *Ras*^*V12*^*; lgl*^*-/-*^ tumor migration tropism through Toll6 [35]. Hence, it is tempting to think that upon the onset of neurogenesis, tumor cells suddenly gain access to elevated levels of Spz concentration from the brain, significantly enhancing local Toll activation and subsequent PGRP-SA and Dorsal expression. This hypothesis may explain the spatial restriction of aberrant Dorsal expression to the posterior part of the tumor.

We have yet to assess whether DlA and DlB exert different functions within *Ras*^*V12*^; *scrib*^*IR*^ tumors, as our knockdowns and overexpression transgenes targeted both isoforms simultaneously. Consistent with previous reports, DlB expression in the tumor context is restricted to cell types from the nervous system. Colocalization of DlB with pJNK in neurons that seem prone to die allows us to speculate if both isoforms have distinct functions, that DlB may promote migration through JNK, while DlA may favor stemness and survival. Additionally, we cannot yet rule out the involvement of other NF-kBs, such as Dif and Relish in *Ras*^*V12*^*; scrib*^*IR*^ tumors.

Using splicing variants may be a mechanism to diversify the range of NF-kB actions in various contexts in *Drosophila*, as it possesses only three NF-kBs (Dorsal, Dif, and Relish). In contrast, mammalian NF-kBs are not reported to have splicing variants, although they possess five different NF-kBs. To our knowledge, the concept of intratumoral NF-kB heterogeneity remains relatively unexplored in mammalian cancer biology and the fly model offer an experimentally accessible model to investigate this question in vivo. Future research efforts will be crucial in elucidating the importance and mechanisms underlying intratumoral NF-kB heterogeneity during malignant transformation.

The *Ras*^*V12*^*, scrib*^*-/-*^ tumor model engages five known inflammatory pathways during transformation. Ras^V12^ directly drives MEK-ERK and PI3K-AKT signaling while engaging Toll-NFkB, whereas loss of cell polarity through scribbled initiates TNFR-JNK MAPK stress signaling and as a result, an Upd/IL6-JAK-STAT autocrine signaling loop altogether favoring malignant transformation.

In conclusion, the findings herein extend the understanding of how innate immune signaling is engaged by and cooperates with oncogenic Ras and opens up for more detailed mechanistic studies in a genetically tractable preclincial tumor model with clear parallels to human cancer.

## Materials and methods

### *Drosophila* lines

The EyMARCM system is known to generate flip-out clones in the adjacent OLs of the CNS in addition to the eye discs [53]. For assessing tumor cell migration from true carcinoma cells arising from the eye disc epithelium, we therefore used another genetic system designed in our lab, called EyaHost, that generates flip-out tumors exclusively in the EAD but not in the CNS [54].

Unless otherwise specified, crosses were conducted at 25°C by default; however, for enhanced knockdown efficiency, most knockdown experiments were carried out at 29°C, as detailed in the figures and legends.

- EyMARCM *Drosophila* lines

Ey,flp; act>STOP>Gal4, UAS-GFP; FRT82B Tub-Gal80ts [34]

Ey,flp, UAS-Dcr2; act>STOP>Gal4, UAS-GFP; FRT82B Tub-Gal80ts;

Sp/CyO, Dfd-YFP; UAS-Ras^V12^ FRT82B scrib^2^/TM6C [55];

UAS-PGRP-SA^IR^; UAS-Ras^V12^ FRT82B scrib^2^/TM6C (BDSC#60,037);

UAS-modSP^IR^; UAS-Ras^V12^ FRT82B scrib^2^/TM6C (VDRC GD#43,972);

UAS-pelle^IR^; UAS-Ras^V12^ FRT82B scrib^2^/TM6C (BDSC#50,715);

UAS-RFP^IR^; UAS-Ras^V12^ FRT82B scrib^2^/TM6C (BDSC#67,852);

UAS-Ras^V12^; FRT82B scrib^1^/TM6C [56];

UAS-Ras^V12^; FRT82B;

FRT82B scrib^1^/TM6C

FRT82B

- EyaHOST *Drosophila* lines

Eya-KD; QUAS-Gal4 (Eya-KD our own lab, QUAS-Gal4 BDSC#83132 [57];

QUAS-Ras^V12^; Eya-KD; QUAS-Gal4 (QUAS-Ras^V12^ from Peter Galant);

QUAS-Ras^V12^; Eya-KD, QUAS-scrib^IR^; QUAS-Gal4 (QUAS-scrib^IR^ from our own lab);

QUAS-Ras^V12^; Eya-KD, QUAS-scrib^IR^;

QUAS-Ras^V12^; Eya-KD, QUAS-scrib^IR^; QUAS-Gal4, UAS-Dcr2 (UAS-Dcr2 BDSC#24651)

Act>|STOP>|QF, QUAS-mCD8::GFP;; (Act>|STOP>|QF from our own lab, QUAS-mCD8::GFP BDSC#30001)

Act>|STOP>|QF, QUAS-mCD8::GFP; UAS-dorsal; (BDSC#9319)

Act>|STOP>|QF, QUAS-mCD8::GFP; UAS-modSP; [58]

Act>|STOP>|QF, QUAS-mCD8::GFP; UAS-diap1^OE^; (BDSC#6657)

Act>|STOP>|QF, QUAS-mCD8::GFP;; UAS-cherry^IR^ (BDSC#35785)

Act>|STOP>|QF, QUAS-mCD8::GFP;; UAS-dorsal^IR-1^ (BDSC#32934)

Act>|STOP>|QF, QUAS-mCD8::GFP;; UAS-dorsal^IR-2^ (BDSC#27650)

Act>|STOP>|QF, QUAS-mCD8::GFP;; UAS-snail^IR^ (VDRC GD#6232)

Act>|STOP>|QF, QUAS-mCD8::GFP;; UAS-twist^IR-1^ (BDSC#51164)

Act>|STOP>|QF, QUAS-mCD8::GFP;; UAS-twist^IR-2^ (BDSC#25981)

Act>|STOP>|QF, QUAS-mCD8::GFP, ey-FLP;; UAS-chinmo^IR-1^ (BDSC#33638)

Act>|STOP>|QF, QUAS-mCD8::GFP, ey-FLP;; QUAS-chinmo^OE^ (BDSC#64777)

Act>|STOP>|QF, QUAS-Tag2BFP;; genomic-dorsal::GFP (BDSC#42677)

Act>|STOP>|QF, QUAS-Tag2BFP;; genomic-cactus::GFP (VDRC fTRG 318,145)

Act>|STOP>|QF, Ey-flp, QUAS- Tag2BFP; TRE-eGFP; (QUAS-Tag2BFP our own lab, TRE-eGFP BDSC#59010)

### Immunohistochemistry

Inverted larvae heads were fixed 30 min in 4% paraformaldehyde/PBS at room temperature on a shaker. After washes, fine dissection of single EADs (for early-stage tumors at Day 6) or whole cephalic complexes (at later stages) was conducted in PBS. The tissues were then incubated with a solution of primary antibody in 0,5% Triton X-100/PBS overnight at 4°C. Noteworthy, for primary antibodies that presented penetration defects (marked with * below), we incubated the tissues in 0,5% Triton X-100/PBS overnight at 4°C prior to primary antibody stainings. Similarly, secondary antibody stainings were done in 0,5% Triton X-100/PBS overnight at 4°C. Samples were then mounted in Vectashield or equivalent mounting media for imaging.

Primary antibodies were used at the following dilutions: anti-DlA mouse antibody (1:50, DSHB 7A4), anti-DlB rabbit antibody (1:1,000, S.Wasserman [32]), anti-pH3 rat (1:200 abcam 10,543), anti-chinmo guinea pig (1:200, Claude Desplan), anti-Elav rat (1:50, DSHB 7E8A10), anti-cleaved Dcp1 rabbit (1:100, Cell Signaling 9,578), anti-phosphoJNK mouse (1:100, Cell Signaling 9,255), and anti-MMP1 mouse (used in a mix 1:1:1, DSHB 3B8D12, DSHB 3A6B4, and DHSB 5H7B11). Secondary antibodies were all from Jackson ImmunoResearch and Thermofisher.

### RNA sequencing and data analysis

Third instar larval eye discs of the indicated genotypes were manually isolated and RNA was extracted using Qiagen RNAmini kits. TruSeq RNA Library Prep Kit (Illumina) was used to prepare libraries, and sequencing was performed on an Illumina MiSeq system. Raw data were analyzed with the Tuxedo suite (RRID:SCR_013194) and reads were mapped to *Drosophila* genome release 5.2. Expression was recorded as FPKM: fragments per kilo-base per million reads (S2 Table). The RNASeq data discussed in this publication have been deposited in NCBI´s Gene Expression Omnibus (GEO) and are accessible through GEO Series accession number GSE291665.

### Imaging

Samples were imaged on Zeiss LSM880 and Zeiss LSM980 confocal microscopes as well as on Nikon HCI SoRa spinning disc microscope. They were further processed on FIJI.

### Tumor volume analysis

Z-stacks were acquired with a 3-µm step for early-stage tumors (Day 6) and a 4-µm step for late-stage tumors (after Day 6). Tumor volume was estimated using a FIJI Macro [59], which automatically performs threshold-based 3D reconstruction of the GFP+ signal, followed by volume measurements (3D Object counter plugin). Before 3D reconstruction, the FIJI Macro applies two consecutive Gaussian blur steps (sigma = 2) to reduce noise.

### Image processing

#### • Fluorescence intensity measurements and heatmaps.

Fluorescence intensity measurements were performed using a FIJI Macro, which subtracts background intensity to the signal of interest inside or outside the tumor, using a tumor mask generated from intensity thresholding. When comparing values across different conditions, they were normalized to the mean value of the control condition, ensuring the control mean is set at 1. In Fig 2B and 2C, the tumors were split in two compartments “high” and “low”, according to arbitrarily set intensity thresholds (250 for DlA, 10,000 for DlB). Normalized mean intensities were then automatically quantified within the different compartments: tumorHigh, tumorLow, and TME. DlA and DlB intensity heatmaps were generated after background subtraction, using the Fire LUT in FIJI.

#### • Colocalization analysis.

Colocalization analyses were performed using a FIJI Macro, which automatically computed the PCC between two channels of a confocal image using the “coloc2” plugin. The PCC, ranging from −1 to 1, denotes the degree of colocalization between signals. A higher absolute value indicates stronger colocalization, with the sign indicating correlation direction (“−” for anti-correlation, “+” for correlation).

#### • Proliferation, differentiation, and apoptosis ratio.

The proliferation, differentiation, and apoptosis ratio are defined as the area of the meaningful marker within the tumor (pH3, Elav, and cDcp, respectively) divided by the area of the tumor. These ratios were measured with a FIJI Macro which measures both the tumor area and the tumor marker area using an automatic-thresholding. For Fig 3A, 3B, and 3C, measurements were conducted on single confocal pictures, whereas, for S1 Fig, the apoptosis ratio was measured on the Z-projection of confocal Z-stacks.

#### • Migration score.

Migration of the VNC and LD was manually scored on Z-projection (Max Intensity mode) of confocal Z-stacks.

### Image analysis with PECAn

For image quantification, we additionally used the recently published PECAn software, which has been specifically designed for clonal analysis [60]. PECAn was used to quantify tumor volumes, marker segmentation (cDcp1 and elav), and fluorescence intensity. The standard tutorial and parameters have been used in the different analyses.

### Statistical analysis

At least, two biological replicates were performed for each experiment. According to the normality and size of the datasets, appropriate statistical tests were applied using GraphPad Prism9 and are indicated in the figure legends. Graphs were also generated on GraphPad Prism9 and are presented as boxplots (10–90 percentile) or dot plots indicating the mean of the datasets. Statistical significance was categorized as follows: *p < 0.05, **p < 0.01, ***p < 0.001, ****p < 0.0001, and n.s. when not significant.

## Supporting information

S1 Fig Localization of DlA, DlB, and Cactus. **(A)** Representative confocal pictures of GFP-labeled *Ras*^*V12*^*; scrib*^*-/-*^ tumors (*n* = 10 EADs) at Day 6, generated with the conventional EyMARCM genetic system and stained for DlA (magenta) and Hoechst (blue). Consistent with the data generated with our new EyaHost genetic system in Fig 2, DlA levels are aberrant in the tumors. The tumor is outlined with a straight white line. Scale bars = 50 µm. **(B)** Representative confocal pictures of GFP-labeled *Ras*^*V12*^*; scrib*^*IR*^ tumors at Day 6, generated with our novel EyaHost genetic system and stained for DlA (green), DlB (magenta), and Hoechst (blue). The tumor cells that already migrated inside the optic lobe of the CNS express high levels of DlA or DlB, suggesting their potential involvement in tumor cell migration. Scale bars = 50 µm. **(C)** Representative confocal pictures of BFP-labeled *genomic-cactus::GFP, Ras*^*V12*^*; scrib*^*IR*^ tumors stained for DlB (magenta) at Day 6 (upper line) (*n* = 6 EADs) or stained for DlA (magenta) at Day 8 (lower line) (*n* = 6 EADs). The tumor is outlined with a straight white line. Scale bars = 20 µm.
(EPS)

S2 Fig The endogenous Dl reporter used in this study is a DlA specific reporter. **(A)** Representative confocal pictures of BFP-labeled *genomic-dorsal::GFP Ras*^*V12*^*; scrib*^*IR*^ tumors at Day 6, stained for DlA (magenta) and DlB (blue). The tumor is outlined with the straight white line. Scale bars = 50 µm. **(B)** Colocalization analyses assessed with the Pearson’s correlation coefficient reveal a good colocalization between DlA and Dorsal::GFP (*n* = 24, *m* = 0,44, SD = ±0,06) and no colocalization between DlB and Dorsal::GFP in the tumors (*n* = 24, *m* = 0,09, SD = ±0,07) suggesting that this reporter is a DlA specific fusion protein. Statistical significance was determined with an unpaired *T* test with Welch’s correction.
(EPS)

S3 Fig Dorsal knockdown efficiency, and pelle knockdown mimics the function of Dorsal on differentiation and survival. **(A)** Representative confocal pictures and quantification of the mean DlA intensity of GFP-labeled *cherry*^*IR*^ *Ras*^*V12*^*; scrib*^*IR*^ (*n* = 6, *m* = 1,00, SD = ±0,14) and *dorsal*^*IR-1*^ *Ras*^*V12*^*; scrib*^*IR*^ tumors (*n* = 6, *m* = 0,54, SD = ±0,03) as well as *Cherry*^*IR*^ *Ras*^*V12*^*; scrib*^*IR*^ *Dcr2* control tumors (*n* = 5, *m* = 1,00, SD = ±0,14) and *dorsal*^*IR-2*^ *Ras*^*V12*^*; scrib*^*IR*^ *Dcr2* tumors (*n* = 6, *m* = 0,32, SD = ±0,05) at Day 6 (29°C). Intensity normalization was done by subtracting the background intensity on each picture and finally dividing each data point by the mean intensity value for the control tumors for each replicate. Statistical significance was determined with a Mann–Whitney test. Scale bars = 20 µm. **(B)** Representative confocal pictures and quantification of the mean DlB intensity of GFP-labeled *cherry*^*IR*^ *Ras*^*V12*^*; scrib*^*IR*^ (*n* = 7, *m* = 1,00, SD = ±0.19) and *dorsal*^*IR-1*^ *Ras*^*V12*^*; scrib*^*IR*^ tumors (*n* = 9, *m* = 0.40, SD = ±0.12) as well as *Cherry*^*IR*^ *Ras*^*V12*^*; scrib*^*IR*^ *Dcr2* control tumors (*n* = 5, *m* = 1.00, SD = ±0.31) and *dorsal*^*IR-2*^ *Ras*^*V12*^*; scrib*^*IR*^ *Dcr2* tumors (*n* = 6, *m* = 0.54, SD = ±0.15) at Day 6 (29°C). Intensity normalization was done by subtracting the background intensity on each picture and finally dividing each data point by the mean intensity value for the control tumors for each replicate. Statistical significance was determined with a Mann–Whitney test. Scale bars = 20 µm. **(C)** Z-projection of representative confocal pictures of GFP-labeled *RFP*^*IR*^ *Ras*^*V12*^*; scrib*^*IR*^ control tumors and *pelle*^*IR*^ *Ras*^*V12*^*; scrib*^*IR*^ tumors at Day 6 (29°C), stained for Elav (green) and cDcp1 (magenta). The tumor is outlined with a straight white line. Quantification of the apoptosis ratio of *RFP*^*IR*^ *Ras*^*V12*^*; scrib*^*IR*^ control tumors (*n* = 16, *m* = 4,97%, SD = ±2,23%) and *pelle*^*IR*^ *Ras*^*V12*^*; scrib*^*IR*^ tumors (*n* = 15, *m* = 17,84%, SD = ±4,16%) at Day 6 (29°C). Here, the apoptosis ratio is defined as the ratio between the cDcp1 area within the tumor and the area of the tumor itself. Statistical significance was determined with an unpaired *T* test with Welch’s correction. Quantification of the differentiation ratio of *RFP*^*IR*^ *Ras*^*V12*^*; scrib*^*IR*^ control tumors (*n* = 5, *m* = 18,04%, SD = ±5,99%) and *pelle*^*IR*^ *Ras*^*V12*^*; scrib*^*IR*^ tumors (*n* = 8, *m* = 44,14%, SD = ±8,83%) at Day 6 (29°C). Here, the differentiation ratio is defined as the ratio between the Elav area within the tumor and the area of the tumor itself. Statistical significance was determined with a Mann–Whitney test. Scale bars = 50 µm.
(EPS)

S4 Fig Dorsal is sufficient to induce dysplasia, delamination, death inhibition, but not overgrowth and invasion. **(A)** Representative confocal pictures and quantification of the mean clone volume of GFP-labeled *wt* clones (*n* = 20, *m* = 5,59 × 10^5^ µm^3^, SD = ±0,69 × 10^5^ µm^3^) and *dorsal*^*OE*^ clones (*n* = 30, *m* = 4,87 × 10^5^ µm^3^, SD = ±0,66 × 10^5^ µm^3^) at Day 6, stained for DlA (magenta) and Hoechst (blue). Dysplastic clones are observed in 100% of the *dorsal*^*OE*^ clones-bearing EADs. GFP-labeled cells are never found in the VNC as indicated with the VNC score values. Scale bars = 50 µm. **(B)** Reconstruction of an orthogonal view of *wt* clones and *dorsal*^*OE*^ clones representing the EAD tissue (magenta) and the GFP-labeled clones (green). GFP-labeled cells extend from the apical to the basal side of the pseudostratified epithelium of the EAD of *wt* clones whereas some *Dorsal*^*OE*^ clones spread basally (yellow arrowhead). **(C)** (left) Representative confocal pictures and quantification of clone elav+ volume coverage for GFP-labeled *wt* clones (*n* = 23, *m* = 45,63, SD = ± 8,418) and *dorsal*^*OE*^ clones (*n* = 23, *m* = 27,27, SD = ±9,166). Images show that part of the cells in *dorsal*^*OE*^ clones do not differentiate and are elav (red) negative post-furrow. Statistical significance was assessed via a Mann–Whitney test; (right) Representative confocal images and quantification of elav+ volume coverage post-furrow for GMR-Gal4 *wt* (*n* = 21, *m* = 26,88, SD = ±4,182) and Dorsal^OE^ EAD (*n* = 12, *m* = 28,27, SD = ±2,994). Images show no change in differentiation status elav+ (red) cells upon post-furrow upregulation of dorsal. The statistical test used was a *t* test with Welch’s correction. Scale bars = 50 µm. **(D)** (left) Representative confocal images and quantification of the density of post-furrow dying cells as measured through Dcp1 cleavage (cDcp1 - red) in GMR-Gal4 *wt* (*n* = 19, *m* = 0,0005874, SD = ± 0,0003230) and Dorsal^OE^ (*n* = 9, *m* = 0,0003489, SD = ± 0,0001982) EAD. Statistical significance was assessed via a Mann–Whitney test. Representative image shows a reduction in cDcp1 cells post furrow upon Dorsal^OE^. Scale bar = 50 µm; (right) Adult *Drosophila* eye of GMR-Gal4 *wt* and Dorsal^OE^.
(EPS)

S5 Fig Snail and Twist genetically contribute to tumor growth. **(A)** Representative confocal pictures and quantification of the mean tumor volume of GFP-labeled *cherry*^*IR*^ *Ras*^*V12*^*; scrib*^*IR*^ (*n* = 12, *m* = 4,83 × 10^5^ µm^3^, SD = ±1,52 × 10^5^ µm^3^) and *snail*^*IR*^ *Ras*^*V12*^*; scrib*^*IR*^ (*n* = 9, *m* = 1,41 × 10^5^ µm^3^, SD = ±0,65 × 10^5^ µm^3^). Statistical significance was determined with a Mann–Whitney’s test. **(B)** Representative confocal pictures and quantification the mean tumor volume of GFP-labeled *cherry*^*IR*^ *Ras*^*V12*^*; scrib*^*IR*^ (*n* = 20, *m* = 7,17 × 10^7^ µm^3^, SD = ±1,22 × 10^7^ µm^3^) and *twist*^*IR-1*^ *Ras*^*V12*^*; scrib*^*IR*^ (*n* = 7, *m* = 1,81 × 10^7^ µm^3^, SD = ±0,83 × 10^7^ µm^3^) as well as *Cherry*^*IR*^ *Ras*^*V12*^*; scrib*^*IR*^ *Dcr2* control tumors (*n* = 24, *m* = 6,89 × 10^7^ µm^3^, SD = ±1,12 × 10^7^ µm^3^) and twist^*IR-2*^ *Ras*^*V12*^*; scrib*^*IR*^ *Dcr2* tumors (*n* = 13, *m* = 3,98 × 10^7^ µm^3^, SD = ±1,43 × 10^7^ µm^3^). VNC invasion scores are indicated. Statistical significance was determined with a Mann–Whitney’s test. Scale bars = 50 µm.
(EPS)

S6 Fig Chinmo is sufficient to induce failure of differentiation, delamination and is induced in *Ras*^*V12*^ *scrib*^*RNAi*^ tumors. **(A)** Representative confocal images and quantification of clone volume and clone elav+ (red) volume coverage for GFP-labeled *wt* (Clone volume: (*n* = 35, *m* = 218,698 µm^3^, SD = ±49,325 µm^3^); Elav+ coverage: (*n* = 35, *m* = 21,14, SD = ± 4,280)) and *chinmo*^*OE*^ clones (Clone volume: (*n* = 30, *m* = 46,875 µm^3^, SD = ±14,343 µm^3^); Elav+ coverage: (*n* = 30, *m* = 3,242, SD = ± 1,774)). Representative images show a reduction in clone volume and a failure of differentiation upon chinmo^OE^. A *t* test with Welch’s correction was used to assess statistical significance for clone volumetrics and a Mann–Whitney test was used to assess statistical significance for clone elav+ volume coverage. Scale bar = 50 µm. **(B)** Orthogonal apical-basal view of the EAD bearing GFP-labeled *wt* clones or chinmo^OE^ clones. A clear delamination behavior can be observed by chinmo^OE^ clones. The EAD nuclei were counterstained with Hoechst (magenta). **(C)** Representative confocal images and quantification of clone chinmo levels (mean gray value) in GFP-labeled *wt Cherry*^*IR*^ *clones* (*n* = 18, *m* = 10,61, SD = ± 1,623), *Cherry*^*IR*^ *Ras*^*V12*^*; scrib*^*IR*^ (*n* = 33, *m* = 23,60, SD = ± 6,810), and *Chinmo*^*IR*^ *Ras*^*V12*^*; scrib*^*IR*^ (*n* = 27, *m* = 14,36, SD = ± 4,821) tumors. Statistical significance was assessed with a Kruskal–Wallis and Dunn’s multiple comparison test. Scale bar = 50 µm.
(EPS)

S1 Table Select list of genes previously reported to have a role in *Drosophila* tumor models alongside Toll pathway components. (PDF)

S2 Table Metadata RNASeq FKPM values. (XLSX)

S3 Table Key resources. (XLSX)

S1 Data (XLSX)

## Abbreviations

CNS: central nervous system
EAD: eye-antennal disc
EMT: Epithelial-mesenchymal transition
GEO: Gene Expression Omnibus
OL: optic lobe
PCC: Pearson’s correlation coefficient
TME: tumor microenvironment
VNC: ventral nerve cord

## References

[1] Hanahan D. Hallmarks of cancer: new dimensions. Cancer Discov. 2022;12(1):31–46. doi: 10.1158/2159-8290.CD-21-1059 35022204

[2] Zhao H, Wu L, Yan G, Chen Y, Zhou M, Wu Y, et al. Inflammation and tumor progression: signaling pathways and targeted intervention. Signal Transduct Target Ther. 2021;6(1):263. doi: 10.1038/s41392-021-00658-5 34248142 PMC8273155

[3] Aggarwal BB, Sung B. NF-κB in cancer: a matter of life and death. Cancer Discov. 2011;1(6):469–71. doi: 10.1158/2159-8290.CD-11-0260 22586649 PMC3392037

[4] Xia L, Tan S, Zhou Y, Lin J, Wang H, Oyang L, et al. Role of the NFκB-signaling pathway in cancer. Onco Targets Ther. 2018;11:2063–73. doi: 10.2147/OTT.S161109 29695914 PMC5905465

[5] Gu L, Wang Z, Zuo J, Li H, Zha L. Prognostic significance of NF-κB expression in non-small cell lung cancer: a meta-analysis. PLoS One. 2018;13(5):e0198223. doi: 10.1371/journal.pone.0198223 29813121 PMC5973575

[6] Sarkar DK, Jana D, Patil PS, Chaudhari KS, Chattopadhyay BK, Chikkala BR, et al. Role of NF-κB as a prognostic marker in breast cancer: a pilot study in indian patients. Indian J Surg Oncol. 2013;4(3):242–7. doi: 10.1007/s13193-013-0234-y 24426730 PMC3771050

[7] Boukerche H, Su Z, Emdad L, Sarkar D, Fisher PB. RETRACTED: mda-9/Syntenin regulates the metastatic phenotype in human melanoma cells by activating nuclear factor-kappaB. Cancer Res. 2007;67(4):1812–22. doi: 10.1158/0008-5472.CAN-06-3875 17308124

[8] Huang S, Pettaway CA, Uehara H, Bucana CD, Fidler IJ. Blockade of NF-kappaB activity in human prostate cancer cells is associated with suppression of angiogenesis, invasion, and metastasis. Oncogene. 2001;20(31):4188–97. doi: 10.1038/sj.onc.1204535 11464285

[9] Huber MA, Azoitei N, Baumann B, Grünert S, Sommer A, Pehamberger H, et al. NF-kappaB is essential for epithelial-mesenchymal transition and metastasis in a model of breast cancer progression. J Clin Invest. 2004;114(4):569–81. doi: 10.1172/JCI21358 15314694 PMC503772

[10] Biswas DK, Shi Q, Baily S, Strickland I, Ghosh S, Pardee AB, et al. NF-kappa B activation in human breast cancer specimens and its role in cell proliferation and apoptosis. Proc Natl Acad Sci U S A. 2004;101(27):10137–42. doi: 10.1073/pnas.0403621101 15220474 PMC454178

[11] Cui X, Shen D, Kong C, Zhang Z, Zeng Y, Lin X, et al. NF-κB suppresses apoptosis and promotes bladder cancer cell proliferation by upregulating survivin expression in vitro and in vivo. Sci Rep. 2017;7:40723. doi: 10.1038/srep40723 28139689 PMC5282527

[12] Talukdar S, Das SK, Pradhan AK, Emdad L, Windle JJ, Sarkar D, et al. MDA-9/Syntenin (SDCBP) is a critical regulator of chemoresistance, survival and stemness in prostate cancer stem cells. Cancers (Basel). 2019;12(1):53. doi: 10.3390/cancers12010053 31878027 PMC7017101

[13] Germani F, Hain D, Sternlicht D, Moreno E, Basler K. The Toll pathway inhibits tissue growth and regulates cell fitness in an infection-dependent manner. Elife. 2018;7:e39939. doi: 10.7554/eLife.39939 30451683 PMC6279345

[14] Meyer SN, Amoyel M, Bergantiños C, de la Cova C, Schertel C, Basler K, et al. An ancient defense system eliminates unfit cells from developing tissues during cell competition. Science. 2014;346(6214):1258236. doi: 10.1126/science.1258236 25477468 PMC5095928

[15] Alpar L, Bergantiños C, Johnston LA. Spatially restricted regulation of spätzle/toll signaling during cell competition. Dev Cell. 2018;46(6):706–719.e5. doi: 10.1016/j.devcel.2018.08.001 30146479 PMC6156939

[16] Bentires-Alj M, Barbu V, Fillet M, Chariot A, Relic B, Jacobs N, et al. NF-kappaB transcription factor induces drug resistance through MDR1 expression in cancer cells. Oncogene. 2003;22(1):90–7. doi: 10.1038/sj.onc.1206056 12527911

[17] Bednarski BK, Ding X, Coombe K, Baldwin AS, Kim HJ. Active roles for inhibitory kappaB kinases alpha and beta in nuclear factor-kappaB-mediated chemoresistance to doxorubicin. Mol Cancer Ther. 2008;7(7):1827–35. doi: 10.1158/1535-7163.MCT-08-0321 18644995 PMC2581801

[18] Xia Y-Z, Ni K, Guo C, Zhang C, Geng Y-D, Wang Z-D, et al. Alopecurone B reverses doxorubicin-resistant human osteosarcoma cell line by inhibiting P-glycoprotein and NF-kappa B signaling. Phytomedicine. 2015;22(3):344–51. doi: 10.1016/j.phymed.2014.12.011 25837271

[19] Baud V, Karin M. Is NF-kappaB a good target for cancer therapy? Hopes and pitfalls. Nat Rev Drug Discov. 2009;8(1):33–40. doi: 10.1038/nrd2781 19116625 PMC2729321

[20] Rasmi RR, Sakthivel KM, Guruvayoorappan C. NF-κB inhibitors in treatment and prevention of lung cancer. Biomed Pharmacother. 2020;130:110569. doi: 10.1016/j.biopha.2020.110569 32750649

[21] Bilder D, Ong K, Hsi T-C, Adiga K, Kim J. Tumour-host interactions through the lens of *Drosophila*. Nat Rev Cancer. 2021;21(11):687–700. doi: 10.1038/s41568-021-00387-5 34389815 PMC8669834

[22] Dillard C, Reis JGT, Rusten TE. RasV12; scrib-/- tumors: a cooperative oncogenesis model fueled by tumor/host interactions. Int J Mol Sci. 2021;22(16):8873. doi: 10.3390/ijms22168873 34445578 PMC8396170

[23] La Marca JE, Richardson HE. Two-faced: roles of JNK signalling during tumourigenesis in the *Drosophila* model. Front Cell Dev Biol. 2020;8:42. doi: 10.3389/fcell.2020.00042 32117973 PMC7012784

[24] Pastor-Pareja JC, Wu M, Xu T. An innate immune response of blood cells to tumors and tissue damage in *Drosophila*. Dis Model Mech. 2008;1(2–3):144–54; discussion 153. doi: 10.1242/dmm.000950 19048077 PMC2562178

[25] Cordero JB, Macagno JP, Stefanatos RK, Strathdee KE, Cagan RL, Vidal M. Oncogenic Ras diverts a host TNF tumor suppressor activity into tumor promoter. Dev Cell. 2010;18(6):999–1011. doi: 10.1016/j.devcel.2010.05.014 20627081 PMC3175220

[26] Khezri R, Holland P, Schoborg TA, Abramovich I, Takáts S, Dillard C, et al. Host autophagy mediates organ wasting and nutrient mobilization for tumor growth. EMBO J. 2021;40(18):e107336. doi: 10.15252/embj.2020107336 34309071 PMC8441431

[27] Holland P, Quintana EM, Khezri R, Schoborg TA, Rusten TE. Computed tomography with segmentation and quantification of individual organs in a *D. melanogaster* tumor model. Sci Rep. 2022;12(1):2056. doi: 10.1038/s41598-022-05991-5 35136137 PMC8825794

[28] Minakhina S, Steward R. Nuclear factor-kappa B pathways in *Drosophila*. Oncogene. 2006;25(51):6749–57. doi: 10.1038/sj.onc.1209940 17072326

[29] Hetru C, Hoffmann JA. NF-kappaB in the immune response of *Drosophila*. Cold Spring Harb Perspect Biol. 2009;1(6):a000232. doi: 10.1101/cshperspect.a000232 20457557 PMC2882123

[30] Valanne S, Wang J-H, Rämet M. The *Drosophila* Toll signaling pathway. J Immunol. 2011;186(2):649–56. doi: 10.4049/jimmunol.1002302 21209287

[31] Gross I, Georgel P, Oertel-Buchheit P, Schnarr M, Reichhart JM. Dorsal-B, a splice variant of the *Drosophila* factor Dorsal, is a novel Rel/NF-kappaB transcriptional activator. Gene. 1999;228(1–2):233–42. doi: 10.1016/s0378-1119(98)00595-2 10072776

[32] Zhou B, Lindsay SA, Wasserman SA. Alternative NF-κB isoforms in the *Drosophila* neuromuscular junction and brain. PLoS One. 2015;10(7):e0132793. doi: 10.1371/journal.pone.0132793 26167685 PMC4500392

[33] Brumby AM, Richardson HE. Scribble mutants cooperate with oncogenic Ras or Notch to cause neoplastic overgrowth in *Drosophila*. EMBO J. 2003;22(21):5769–79. doi: 10.1093/emboj/cdg548 14592975 PMC275405

[34] Pagliarini RA, Xu T. A genetic screen in *Drosophila* for metastatic behavior. Science. 2003;302(5648):1227–31. doi: 10.1126/science.1088474 14551319

[35] Mishra-Gorur K, Li D, Ma X, Yarman Y, Xue L, Xu T. Spz/Toll-6 signal guides organotropic metastasis in *Drosophila*. Dis Model Mech. 2019;12(10):dmm039727. doi: 10.1242/dmm.039727 31477571 PMC6826028

[36] De Gregorio E, Spellman PT, Tzou P, Rubin GM, Lemaitre B. The Toll and Imd pathways are the major regulators of the immune response in *Drosophila*. EMBO J. 2002;21(11):2568–79. doi: 10.1093/emboj/21.11.2568 12032070 PMC126042

[37] Scott ML, Fujita T, Liou HC, Nolan GP, Baltimore D. The p65 subunit of NF-kappa B regulates I kappa B by two distinct mechanisms. Genes Dev. 1993;7(7A):1266–76. doi: 10.1101/gad.7.7a.1266 8319912

[38] Ghosh S, May MJ, Kopp EB. NF-kappa B and Rel proteins: evolutionarily conserved mediators of immune responses. Annu Rev Immunol. 1998;16:225–60. doi: 10.1146/annurev.immunol.16.1.225 9597130

[39] Külshammer E, Mundorf J, Kilinc M, Frommolt P, Wagle P, Uhlirova M. Interplay among *Drosophila* transcription factors Ets21c, Fos and Ftz-F1 drives JNK-mediated tumor malignancy. Dis Model Mech. 2015;8(10):1279–93. doi: 10.1242/dmm.020719 26398940 PMC4610234

[40] Uhlirova M, Bohmann D. JNK- and Fos-regulated Mmp1 expression cooperates with Ras to induce invasive tumors in *Drosophila*. EMBO J. 2006;25(22):5294–304. doi: 10.1038/sj.emboj.7601401 17082773 PMC1636619

[41] Srivastava A, Pastor-Pareja JC, Igaki T, Pagliarini R, Xu T. Basement membrane remodeling is essential for *Drosophila* disc eversion and tumor invasion. Proc Natl Acad Sci USA. 2007;104(8):2721–6. doi: 10.1073/pnas.0611666104 17301221 PMC1815248

[42] Külshammer E, Uhlirova M. The actin cross-linker Filamin/Cheerio mediates tumor malignancy downstream of JNK signaling. J Cell Sci. 2013;126(Pt 4):927–38. doi: 10.1242/jcs.114462 23239028

[43] Doggett K, Turkel N, Willoughby LF, Ellul J, Murray MJ, Richardson HE, et al. BTB-Zinc finger oncogenes are required for ras and notch-driven tumorigenesis in *Drosophila*. PLoS One. 2015;10(7):e0132987. doi: 10.1371/journal.pone.0132987 26207831 PMC4514741

[44] Wu C, Ding X, Li Z, Huang Y, Xu Q, Zou R, et al. CtBP modulates Snail-mediated tumor invasion in *Drosophila*. Cell Death Discov. 2021;7(1):202. doi: 10.1038/s41420-021-00516-x 34349099 PMC8339073

[45] Jiang J, Kosman D, Ip YT, Levine M. The dorsal morphogen gradient regulates the mesoderm determinant twist in early *Drosophila* embryos. Genes Dev. 1991;5(10):1881–91. doi: 10.1101/gad.5.10.1881 1655572

[46] Ip YT, Park RE, Kosman D, Yazdanbakhsh K, Levine M. dorsal-twist interactions establish snail expression in the presumptive mesoderm of the *Drosophila* embryo. Genes Dev. 1992;6(8):1518–30. doi: 10.1101/gad.6.8.1518 1644293

[47] Stathopoulos A, Levine M. Whole-genome analysis of *Drosophila* gastrulation. Curr Opin Genet Dev. 2004;14(5):477–84. doi: 10.1016/j.gde.2004.07.004 15380237

[48] Hong J-W, Hendrix DA, Papatsenko D, Levine MS. How the Dorsal gradient works: insights from postgenome technologies. Proc Natl Acad Sci USA. 2008;105(51):20072–6. doi: 10.1073/pnas.0806476105 19104040 PMC2629255

[49] Enomoto M, Takemoto D, Igaki T. Interaction between Ras and Src clones causes interdependent tumor malignancy via Notch signaling in *Drosophila*. Dev Cell. 2021;56(15):2223-2236.e5. doi: 10.1016/j.devcel.2021.07.002 34324859

[50] Jayawant E, Pack A, Clark H, Kennedy E, Ghodke A, Jones J, et al. NF-κB fingerprinting reveals heterogeneous NF-κB composition in diffuse large B-cell lymphoma. Front Oncol. 2023;13:1181660. doi: 10.3389/fonc.2023.1181660 37333821 PMC10272839

[51] Siddiqui I, Erreni M, Kamal MA, Porta C, Marchesi F, Pesce S, et al. Differential role of Interleukin-1 and Interleukin-6 in K-Ras-driven pancreatic carcinoma undergoing mesenchymal transition. Oncoimmunology. 2017;7(2):e1388485. doi: 10.1080/2162402X.2017.1388485 29308316 PMC5749654

[52] Treisman JE. Retinal differentiation in *Drosophila*. Wiley Interdiscip Rev Dev Biol. 2013;2(4):545–57. doi: 10.1002/wdev.100 24014422 PMC3909661

[53] Newsome TP, Asling B, Dickson BJ. Analysis of *Drosophila* photoreceptor axon guidance in eye-specific mosaics. Development. 2000;127(4):851–60. doi: 10.1242/dev.127.4.851 10648243

[54] Teles-Reis J, Jain A, Liu D, Khezri R, Micheli S, Gomez AA, et al. EyaHOST, a modular genetic system for investigation of intercellular and tumor-host interactions in *Drosophila melanogaster*. bioRxiv. 2024:2024.09.06.611647. doi: 10.1101/2024.09.06.611647 39314415 PMC11418954

[55] Bilder D, Perrimon N. Localization of apical epithelial determinants by the basolateral PDZ protein Scribble. Nature. 2000;403(6770):676–80. doi: 10.1038/35001108 10688207

[56] Wu M, Pastor-Pareja JC, Xu T. Interaction between Ras(V12) and scribbled clones induces tumour growth and invasion. Nature. 2010;463(7280):545–8. doi: 10.1038/nature08702 20072127 PMC2835536

[57] Baena-Lopez LA, Arthurton L, Bischoff M, Vincent J-P, Alexandre C, McGregor R. Novel initiator caspase reporters uncover previously unknown features of caspase-activating cells. Development. 2018;145(23):dev170811. doi: 10.1242/dev.170811 30413561 PMC6288387

[58] Dostálová A, Rommelaere S, Poidevin M, Lemaitre B. Thioester-containing proteins regulate the Toll pathway and play a role in *Drosophila* defence against microbial pathogens and parasitoid wasps. BMC Biol. 2017;15(1):79. doi: 10.1186/s12915-017-0408-0 28874153 PMC5584532

[59] Schindelin J, Arganda-Carreras I, Frise E, Kaynig V, Longair M, Pietzsch T, et al. Fiji: an open-source platform for biological-image analysis. Nat Methods. 2012;9(7):676–82. doi: 10.1038/nmeth.2019 22743772 PMC3855844

[60] Baumgartner ME, Langton PF, Logeay R, Mastrogiannopoulos A, Nilsson-Takeuchi A, Kucinski I, et al. The PECAn image and statistical analysis pipeline identifies minute cell competition genes and features. Nat Commun. 2023;14(1):2686. doi: 10.1038/s41467-023-38287-x 37164982 PMC10172353

