# Supplementary material for: NF-κB signaling driven by oncogenic Ras contributes to tumorigenesis in a Drosophila carcinoma model
Source: PLoS Biol. 2025 Apr 28;23(4):e3002663. doi: 10.1371/journal.pbio.3002663 (PMC12037074; doi:10.1371/journal.pbio.3002663)
Supplement: S1 Table — (PDF) [file pbio.3002663.s007.pdf]

Table 1. Select list of genes previously reported to have a role in *Drosophila* tumor models alongside Toll pathway components

FlyBase_ID	Symbol	RasV12, Scrib- GFP+	GMR GFP+ Control	FC	Category	References
FBgn0035379	spz5	6,8	0,3	22,37932864	Immunity	
FBgn0000533	ea	20,8	2,2	9,598286464	Immunity	
FBgn0036690	Ilp8	692,7	99,8	6,942235648	Classic	[1]
FBgn0030964	Pvf1	31,6	5,5	5,729703098	Classic	[2]
FBgn0014141	cher	188,0	33,2	5,660521626	Classic	[3]
FBgn0001257	ImpL2	1246,3	231,7	5,378162502	Classic	[4] [5]
FBgn0004583	ex	232,4	46,0	5,054412964	Classic	[6] [7]
FBgn0004009	wg	29,5	7,1	4,149265269	Classic	[8]
FBgn0031959	spz3	59,5	15,4	3,849215853	Immunity	
FBgn0005660	Ets21C	58,4	15,7	3,727326075	Classic	[9]
FBgn0014135	bnl	9,6	2,7	3,522002778	Classic	[10]
FBgn0030310	PGRP-SA	56,2	16,4	3,433538657	Immunity	
FBgn0040322	GNBP2	14,0	4,7	3,008103429	Immunity	
FBgn0043903	dome	51,8	19,1	2,711640253	Classic	[11]
FBgn0035023	itp	76,3	28,1	2,710168154	Classic	[12]
FBgn0035049	Mmp1	285,2	106,1	2,688957819	Classic	[13]
FBgn0041092	tai	37,1	14,4	2,578619411	Classic	[9]
FBgn0051217	modSP	30,6	12,0	2,547377186	Immunity	
FBgn0001078	ftz-f1	24,4	9,7	2,524185474	Classic	[14] [9]
FBgn0030904	upd2	14,8	7,5	1,990392043	Classic	[11]

Select RNASeq read counts and fold change above ~2 between Ras^{V12}, $scrib^{-/-}$ GFP+ tumor cells and control GmrGal4,UAS-GFP cells. Classic: Previously identified genes known to have a role in *Drosophila* tumour growth, invasion, or Tumour-Host interactions. Upregulated Toll pathway genes are indicated in grey.

References

1. Colombani J, Andersen DS, Léopold P. Secreted peptide Dilp8 coordinates Drosophila tissue growth with developmental timing. Science. 2012;336(6081):582-5. doi: 10.1126/science.1216689. PubMed PMID: 22556251.
2. Song W, Kir S, Hong S, Hu Y, Wang X, Binari R, et al. Tumor-Derived Ligands Trigger Tumor Growth and Host Wasting via Differential MEK Activation. Dev Cell. 2019;48(2):277-86.e6. Epub 20190110. doi: 10.1016/j.devcel.2018.12.003. PubMed PMID: 30639055; PubMed Central PMCID: PMCPMC6368352.
3. Kulshammer E, Uhlirova M. The actin cross-linker Filamin/Cheerio mediates tumor malignancy downstream of JNK signaling. J Cell Sci. 2013;126(Pt 4):927-38. Epub 20121213. doi: 10.1242/jcs.114462. PubMed PMID: 23239028.
4. Kwon Y, Song W, Droujinine IA, Hu Y, Asara JM, Perrimon N. Systemic organ wasting induced by localized expression of the secreted insulin/IGF antagonist ImpL2. Dev Cell. 2015;33(1):36-46. doi: 10.1016/j.devcel.2015.02.012. PubMed PMID: 25850671; PubMed Central PMCID: PMCPMC4437243.
5. Figueroa-Clarevega A, Bilder D. Malignant Drosophila tumors interrupt insulin signaling to induce cachexia-like wasting. Dev Cell. 2015;33(1):47-55. doi: 10.1016/j.devcel.2015.03.001. PubMed PMID: 25850672; PubMed Central PMCID: PMCPMC4390765.
6. Doggett K, Grusche FA, Richardson HE, Brumby AM. Loss of the Drosophila cell polarity regulator Scribbled promotes epithelial tissue overgrowth and cooperation with oncogenic Ras-Raf through impaired Hippo pathway signaling. BMC developmental biology. 2011;11:57. Epub 20110929. doi: 10.1186/1471-213x-11-57. PubMed PMID: 21955824; PubMed Central PMCID: PMCPMC3206446.
7. Sun G, Irvine KD. Regulation of Hippo signaling by Jun kinase signaling during compensatory cell proliferation and regeneration, and in neoplastic tumors. Dev Biol. 2011;350(1):139-51. Epub 20101209. doi: 10.1016/j.ydbio.2010.11.036. PubMed PMID: 21145886; PubMed Central PMCID: PMCPMC3038240.
8. Lee J, Ng KG, Dombek KM, Eom DS, Kwon YV. Tumors overcome the action of the wasting factor ImpL2 by locally elevating Wnt/Wingless. Proceedings of the National Academy of Sciences of the United States of America. 2021;118(23). doi: 10.1073/pnas.2020120118. PubMed PMID: 34078667; PubMed Central PMCID: PMCPMC8201939.
9. Atkins M, Potier D, Romanelli L, Jacobs J, Mach J, Hamaratoglu F, et al. An Ectopic Network of Transcription Factors Regulated by Hippo Signaling Drives Growth and Invasion of a Malignant Tumor Model. Curr Biol. 2016;26(16):2101-13. Epub 20160728. doi: 10.1016/j.cub.2016.06.035. PubMed PMID: 27476594.

10. Newton H, Wang Y-F, Camplese L, Mokochinski JB, Kramer HB, Brown AEX, et al. Systemic muscle wasting and coordinated tumour response drive tumourigenesis. Nature Communications. 2020;11(1):4653. doi: 10.1038/s41467-020-18502-9.
11. Wu M, Pastor-Pareja JC, Xu T. Interaction between RasV12 and scribbled clones induces tumour growth and invasion. Nature. 2010;463(7280):545-8. doi: 10.1038/nature08702.
12. Xu W, Li G, Chen Y, Ye X, Song W. A novel antidiuretic hormone governs tumour-induced renal dysfunction. Nature. 2023;624(7991):425-32. doi: 10.1038/s41586-023-06833-8.
13. Uhlirova M, Bohmann D. JNK- and Fos-regulated Mmp1 expression cooperates with Ras to induce invasive tumors in Drosophila. EMBO J. 2006;25(22):5294-304. Epub 20061102. doi: 10.1038/sj.emboj.7601401. PubMed PMID: 17082773; PubMed Central PMCID: PMCPMC1636619.
14. Kulshammer E, Mundorf J, Kilinc M, Frommolt P, Wagle P, Uhlirova M. Interplay among Drosophila transcription factors Ets21c, Fos and Ftz-F1 drives JNK-mediated tumor malignancy. Dis Model Mech. 2015;8(10):1279-93. Epub 20150806. doi: 10.1242/dmm.020719. PubMed PMID: 26398940; PubMed Central PMCID: PMCPMC4610234.
